# Supramolecular hydration structure of graphene-based hydrogels: density functional theory, green chemistry and interface application

**DOI:** 10.3762/bjnano.16.61

**Published:** 2025-06-04

**Authors:** Hon Nhien Le, Duy Khanh Nguyen, Minh Triet Dang, Huyen Trinh Nguyen, Thi Bang Tam Dao, Trung Do Nguyen, Chi Nhan Ha Thuc, Van Hieu Le

**Affiliations:** 1 Faculty of Materials Science and Technology, University of Science, Ho Chi Minh City, 700000, Vietnam; 2 Vietnam National University, Ho Chi Minh City, 700000, Vietnam; 3 Laboratory for Computational Physics, Institute for Computational Science and Artificial Intelligence, Van Lang University, Ho Chi Minh City, 700000, Vietnam; 4 Faculty of Mechanical – Electrical and Computer Engineering, School of Technology, Van Lang University, Ho Chi Minh City, 700000, Vietnam; 5 School of Education, Can Tho University, Can Tho City, 90000, Vietnam; 6 Faculty of Chemistry, University of Science, Ho Chi Minh City, 700000, Vietnam; 7 Multifunctional Materials Laboratory, University of Science, Ho Chi Minh City, 700000, Vietnam

**Keywords:** antibacterial coating, bioinspired hydration, density functional theory, graphene-based hydrogel, supramolecular structure

## Abstract

Natural hydration shells are discovered to play an essential role in the structure and function of biomolecules (deoxyribonucleic acid, protein, and phospholipid membrane). Hydration layers are also important to the structure and property of artificial graphene-based materials. Our recent works prove that graphene-based hydrogels are supramolecular hydration structures that preserve graphene nanosheets from the restacking through hydrophobic force, van der Waals force, and π–π interaction. In this manuscript, density functional theory and high-performance computing (HPC) are used for modeling and calculating van der Waals force between graphene nanosheets in water-intercalated AB bilayer graphene structures. A layer of water molecules significantly decreases the intersheet van der Waals force. A novel hydrogel of graphene oxide–silica gel–zinc hydroxide (GO-SG-ZH) is experimentally synthesized to demonstrate the advantages of hydrated hydrogel structure in comparison with dry powder structure. The synthesis of graphene-based hydrogels is a green chemistry approach to attain extraordinary properties of graphene-based nanostructures. Analytical characterizations exhibited moisture contents, water evaporation rates, three-dimensional structures, elemental compositions, aqueous dispersibility, and antibacterial activities. Hydration shells on graphene-based nanosheets in the hydrogel increase intersheet distances to prevent the stacking of the nanostructures. Hydration layers in the GO-SG-ZH hydrogel was also lubricative for direct brush coating on polymer substrates, typically polylactide films. Interfacial adhesion of graphene-based nanosheets on polylactide substrates made the antibacterial coating stable for several application purposes. In general, supramolecular graphene-based hydrogels are bioinspired hydration structures to advance nanoscale properties and nanotechnology applications.

## Introduction

Biological cells are assemblies of biomolecules that are hydrated with water molecules. The cell content includes about 70–95% water that creates an aqueous environment for biological processes. Water molecules are bound to biomolecular surfaces and participate in the structuring and functioning of biomolecules, typically the folding of protein and the twisting of the double helix of deoxyribonucleic acid (DNA) [1]. Water molecules and their hydrogen bonding network function as lubricants for biomolecular dynamics. Recent scientific works have analyzed the important role of hydration shells on DNA, proteins, and phospholipid membranes [2–4]. The first hydration shell (about 3.5 Å) at the interface of biomolecules has considerably slower dynamics than water molecules in the bulk. Besides, the first water layer on the interface is responsible for hydration forces between biomolecular structures [5]. The rearrangement of water molecules through hydrogen bonding on hydrated surfaces generates repulsive hydration forces when another surface perturbs the hydration layers [6–8]. Hydration shells and hydration forces keep the hydrated structures stable and functional in the natural concert of biological processes.

In the aspect of artificial nanomaterials, it is proposed that hydration also plays an important role in the stability and functionality of nanoscale structures. Van der Waals forces are supramolecular intermolecular interactions that govern the agglomeration of nanomaterials. Carbon nanostructures with π-conjugated systems (fullerene, carbon nanotube, and graphene) have π–π interactions, a type of van der Waals force, for supramolecular attraction [9]. Particularly, graphene sheets with a large surface area and π-conjugated network are likely to stack together through hydrophobic agglomeration and π–π interaction. Although π–π interactions are generally weaker than hydrogen bonding, two graphene sheets in face-to-face geometry have a large interaction surface area to multiply the van der Waals force per unit area, resulting in strong binding energy of total attraction forces. The restacking of graphene-based nanosheets, including pristine graphene, graphene oxide (GO), and reduced graphene oxide (RGO), causes the drawbacks of small effective surface area and low dispersibility in media [10]. Several approaches have been reported to prevent the irreversible stacking of graphene-based nanosheets, including electrostatic repulsion, nanoparticle intercalation, three-dimensional assembly, and surface hydration [10–12]. In our previous works, a number of graphene-based hydrogels (RGO-SnO_2_, RGO-ZnO, and RGO) were synthesized to evidence the reversible self-assembly of graphene-based nanosheets thanks to water intercalation in the hydrated ensembles [13–17]. Therefore, supramolecular graphene-based hydrogels with hydration intercalation and hydration force are quite useful for preserving and generating graphene-based nanosheets for many applications.

In this manuscript, we calculated van der Waals forces in bilayer graphene structures using density functional theory modeling (DFT) and dispersion energy correction functional (DFT-D3). The theoretical work aimed to elucidate the relationship between water intercalation and intersheet binding energy in quantum mechanical level. The computational calculations quantified intersheet distance, van der Waals force, bandgap energy, and formation energy of the molecular system of bilayer graphene intercalated with a water layer. In the experimental aspect, green chemistry methods were applied for synthesizing GO nanosheets, rice-husk-derived silica gel (SG), nanosilica–zinc hydroxide nanoparticles (SG-ZH), and graphene oxide–nanosilica–zinc hydroxide nanocomposites (GO-SG-ZH). Graphite oxidation reaction in a cascade design gives good efficiency values of energy, chemical reaction, and reaction time [14–15]. The recycling of rice husk ash waste into nanosilica products is eco-friendly and sustainable for circular economy [18–21]. Especially, GO nanosheets decorated with SG-ZH nanoparticles have hydrophilic surfaces to retain hydration layers in the hydrogel structure of the GO-SG-ZH nanocomposite. Hydration layers in the GO-SG-ZH hydrogel also function as lubricants at the nanomaterials interfaces, leading to facile brush coating on plastic films of polylactide (PLA). Dehydrated GO-SG-ZH coating is adhered to the PLA substrate through interfacial interactions. Furthermore, antibacterial activities, coating stability, and mechanical properties of the nanocomposite materials were investigated and described in the results and discussion.

## Methods

### Computation method of density functional theory

First-principles calculations based on DFT were conducted using the Vienna ab initio simulation software (VASP) and a high-performance computing system (HPC). The projector-augmented wave method (PAW) was implemented in electronic structure calculations. Generalized gradient approximation of Perdew–Burke–Ernzerhof (GGA-PBE) was used for describing exchange-correlation energy of electron–electron interactions. The correction of van der Waals dispersion energy was applied using the DFT-D3 method proposed by Grimme [22–24]. The modeling of infinite graphene sheets was extrapolated from periodic supercells. The supercell of bilayer graphene structure includes 16 carbon atoms (two graphene sheets with eight carbon atoms per sheet). The modeling of water-intercalated bilayer graphene structure used the supercell of 16 carbon atoms, one oxygen atom, and two hydrogen atoms (two graphene sheets and one water molecule).

#### Preparation of graphene oxide from natural graphite

The improved cascade-design synthesis of graphite oxide (GrO) was reported in our previous papers [15–16]. Briefly, 5 g of raw material of natural graphite (Shanghai Zhanyun Chemical) was soaked and agitated in 50 mL of 98% sulfuric acid. The solution of Mn(VII) compound was prepared by dissolving 10 g of potassium permanganate in 100 mL of 98% sulfuric acid. The graphite/H_2_SO_4_ suspension was slowly poured into the Mn(VII) solution. A cooling water bath and an infrared thermometer were used for controlling the reactor temperature below 55 °C (the peak of the reactor temperature is about 50 °C). After agitation in room-temperature conditions for 4 h, the graphite/Mn(VII)/H_2_SO_4_ suspension was slowly poured into 360 mL of water (the exothermic heat increased the reactor temperature to above 90 °C). After 2 h of agitation, the reaction was mixed with 150 mL of a 5% H_2_O_2_ solution and kept stirring for one day. After washing to neutral pH, the material was dried and ground to produce a GrO powder (moisture ≈20%). Next, the GrO powder was dispersed in water and sonicated for 1 h. After natural sedimentation overnight, the suspension was decanted to collect the supernatant dispersion of GO nanosheets.

#### Preparation of nanosilica from rice husk ash waste

Rice husk ash that was discarded from industrial boilers was collected for recycling experiments. Our method of nanosilica synthesis using potassium hydroxide and acetic acid was mentioned in a recent paper [21]. Raw material from rice husk ash waste was dispersed in a 7% potassium hydroxide solution. The suspension was agitated for 1 h at a temperature range of 80–90 °C. After careful filtration, a clear yellow solution of potassium silicate was obtained and neutralized with a 15% acetic acid solution. After that, the suspension of precipitated nanosilica was incubated overnight and then thoroughly washed with water. The obtained product of silica gel (SG) was used for subsequent synthesis and brush coating experiments.

#### Synthesis of graphene oxide–nanosilica–zinc hydroxide hydrogel

The suspension of 0.625 g GrO and 250 mL of water was agitated and then sonicated for 1 h. The suspension was decanted to collect about 250 mL of GO dispersion. An amount of 4.4 g of Zn(CH_3_COO)_2_·2H_2_O was dissolved in 250 mL of water to prepare a 250 mL Zn^2+^ solution. The Zn^2+^ solution was slowly dropped into the GO solution under stirring. The obtained GO/Zn^2+^ dispersion was sonicated for 30 min. Besides, 20 g of the SG material (moisture ≈95%) was mechanically dispersed in 480 mL of water for 15 min and then sonicated for 15 min. A volume of 500 mL of the as-prepared GO/Zn^2+^ dispersion was dropped into the 500 mL SG dispersion. The mixture was agitated for 15 min and sonicated for 15 min. Then, the reaction was adjusted to pH 10 using ammonia solution for Zn(OH)_2_ precipitation and kept stirring for 1 h. After sedimentation, the material was filtered and thoroughly washed with water. A hydrogel of graphene oxide–nanosilica–zinc hydroxide (GO-SG-ZH hydrogel) was collected and analyzed.

To produce a GO-SG-ZH product in powder form, the GO-SG-ZH hydrogel was dried at 80 °C and ground to obtain the GO-SG-ZH powder. The graphene-based nanocomposites in hydrogel form and in powder form were comparatively characterized using moisture analysis, scanning electron microscopy (SEM), energy-dispersive X-ray spectroscopy (EDS), and aqueous dispersibility.

#### Brush coating of graphene oxide–nanosilica–zinc hydroxide hydrogel on polylactide film

At first, commercial polylactide granules (PLA Luminy LX175, TotalEnergies Corbion) was put in a steel mold for thermal compression at 190 °C to produce a PLA plate. A piece of the PLA plate was put in a thin plastic mold (polyethylene terephthalate) for thermal compression at 190 °C. As a result, thin and transparent PLA films were made with the average thickness of 0.2 mm.

In the next stage, the GO-SG-ZH nanocomposite in hydrogel form (≈95% water) was used as an aqueous paint for brush coating on PLA thin films. After brush coating, the coated films were left to air dry for 3 h and were mildly dried using a hair dryer. The obtained coated films were denoted as GO-SG-ZH/PLA. Besides, the as-synthesized SG hydrogel (≈95% water) was also suitable for direct brush coating on PLA films. A similar procedure of brush coating was applied to produce PLA thin films coated with nanosilica. The nanosilica-coated films were denoted as SG/PLA.

#### Materials characterization

Materials weight and moisture values were measured using a laboratory balance (Ohaus Pioneer, 220 g/0.0001 g) and a moisture analyzer (A&D Weighing MX-50, 51 g/0.001 g), respectively. Scanning electron microscopy and energy-dispersive X-ray spectroscopy were performed using a JSM-IT200 system (JEOL). Samples were coated with Pt before the SEM-EDS analysis. X-ray diffraction was performed on a D8 Advance instrument (Bruker). Fourier-transform infrared spectroscopy (FTIR) was characterized with a FT/IR-6600 instrument (Jasco). Ultraviolet–visible absorption spectroscopy (UV–vis) and light transmittance spectroscopy were recorded using a V-670 spectrophotometer (Jasco). Microscopic texture and imaging were observed by a stereo zoom microscope (Optika SZM). Agar diffusion assays were used for testing antibacterial activity against *E. coli* and *S. aureus* (the positive control was the antibiotic penicillin). Inhibition zone assays were used to evaluate antibiofilm properties of uncoated and coated plastic films [14,25]. Coating stability of plastic films in an environment simulating aqueous food was tested using the method reported in our previous paper [14,26]. Measurement of tensile properties was conducted using a universal tensile testing machine (Yang Yi Technology, 500-N load cell) and the ASTM D882-18 standard [14,27].

## Results and Discussion

### Density functional theory calculations of intersheet distances, van der Waals forces and bandgaps

The van der Waals force between two graphene nanosheets arises from the π–π interaction between π orbitals of carbon atoms in one graphene sheet and π orbitals of carbon atoms in the other graphene sheet. The van der Waals force is responsible for AB graphene stacking in natural multilayer graphite [28]. Production processes convert multilayer graphite into single-layer graphene sheets dispersed in solvent medium. However, after the drying process, solvation shells of graphene sheets are removed, resulting in smaller distances between graphene sheets and larger interaction surfaces. With a short intersheet distance and large interaction surface area, van der Waals forces between graphene sheets increase to a higher binding energy which accounts for the restacking of graphene sheets. The restacking of graphene materials at dry state is the main cause of lower aqueous dispersibility and reduced surface area of graphene-based materials in many applications, such as aqueous dispersions, polymer nanocomposites, and water-based paints. Our previous works demonstrated that in graphene-based hydrogel structures, the intercalation of water molecules between graphene-based sheets maintains large intersheet distances and low interaction surface area, which leads to reduced binding energy of van der Waals force [14–15]. The simple method of water intercalation in hydrogel structures is an effective bioinspired approach to prevent nanosheet stacking and preserve graphene-based nanostructures.

**Figure 1:**
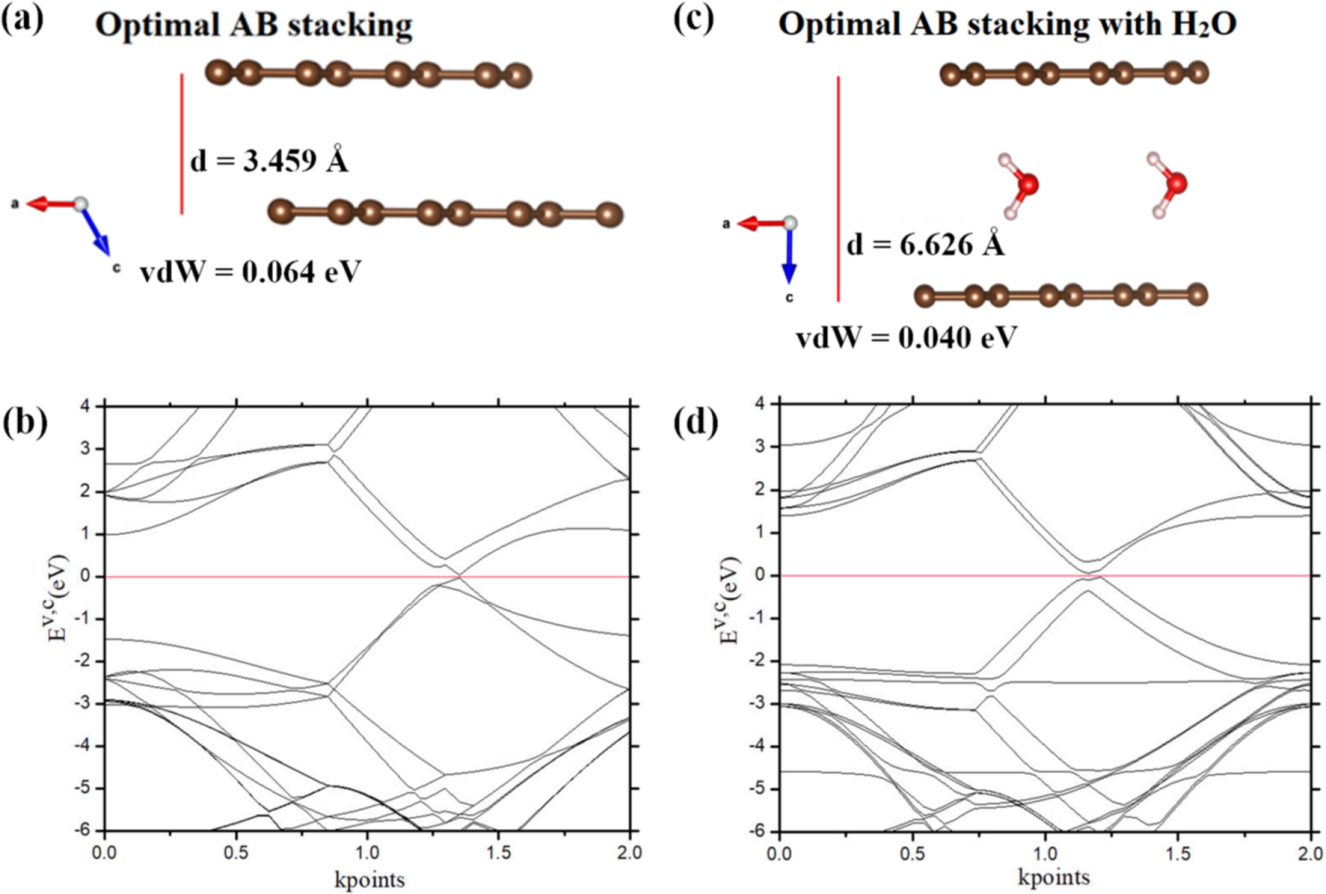
DFT modelling of AB bilayer graphene structures. The AB bilayer graphene with the intersheet distance of 3.459 Å (a), and its valence–conduction band structure in hexagonal Brillouin zone (b). The AB bilayer graphene structure intercalated with a layer of water molecules and the intersheet distance of 6.626 Å (c), and its electronic band structure in hexagonal Brillouin zone (d).

Herein, DFT calculations were performed to quantify the van der Waals dispersion interactions in pristine bilayer graphene and water-intercalated bilayer graphene structures. In natural multilayer graphite, graphene sheets stack together in an AB configuration. Figure 1a shows the modeling of a bilayer graphene structure that mimics the AB stacking in multilayer graphite. DFT optimization calculation presented that pristine bilayer graphene has the formation energy of −9.3778 eV/supercell, intersheet distance of 3.459 Å, and van der Waals binding energy of 0.064 eV/atom (Figure 1a and Table 1). The intersheet distance is comparable to the values reported in other papers [29–31]. The bilayer graphene structure has a small bandgap of 0.06 eV which is slightly open in comparison to the zero bandgap of a single-layer graphene sheet.

**Table 1:** Formation energies, intersheet distances, van der Waals forces, and band gaps of bilayer graphene structures.

Configuration	Formation energy (eV)	C–C bond length (Å)	Intersheet distance (Å)	van der Waals force (eV)	Bandgap (eV)

Pristine AB bilayer graphene	−9.3778	1.42	3.459	0.064	0.06
AB bilayer graphene intercalated with a water layer	−10.6414	1.42	6.626	0.040	0.09

Besides, DFT modeling of the water-intercalated AB bilayer graphene structure was also calculated by HPC. The resulting formation energy is −10.6414 eV/supercell (Table 1). In the optimal structure (Figure 1c and 1d), hydrogen atoms of the water molecule are oriented toward graphene sheets due to the hydrogen–π interaction. It is noteworthy that the enlarged intersheet distance of 6.626 Å led to the intersheet binding energy of 0.04 eV/atom. A layer of water molecules in between two graphene sheets significantly declined the van der Waals force by 37.5% (from 0.064 to 0.040 eV). The bandgap of the water-intercalated bilayer graphene structure increased to 0.09 eV. Although the opening of the bandgap is still small, it is suggested that the bandgap of the AB bilayer graphene can be further opened by increasing molecular water layers in the intersheet spacing as well as the spacing distance. The approach of water intercalation in graphene-based structures is effective for lowering van der Waals force and opening the bandgap. Therefore, water-intercalated structures of graphene-based nanosheets should be experimentally synthesized to ameliorate the nanostructures and properties for various applications in science and industry.

### Supramolecular hydration structure of graphene oxide–nanosilica–zinc hydroxide hydrogel

**Figure 2:**
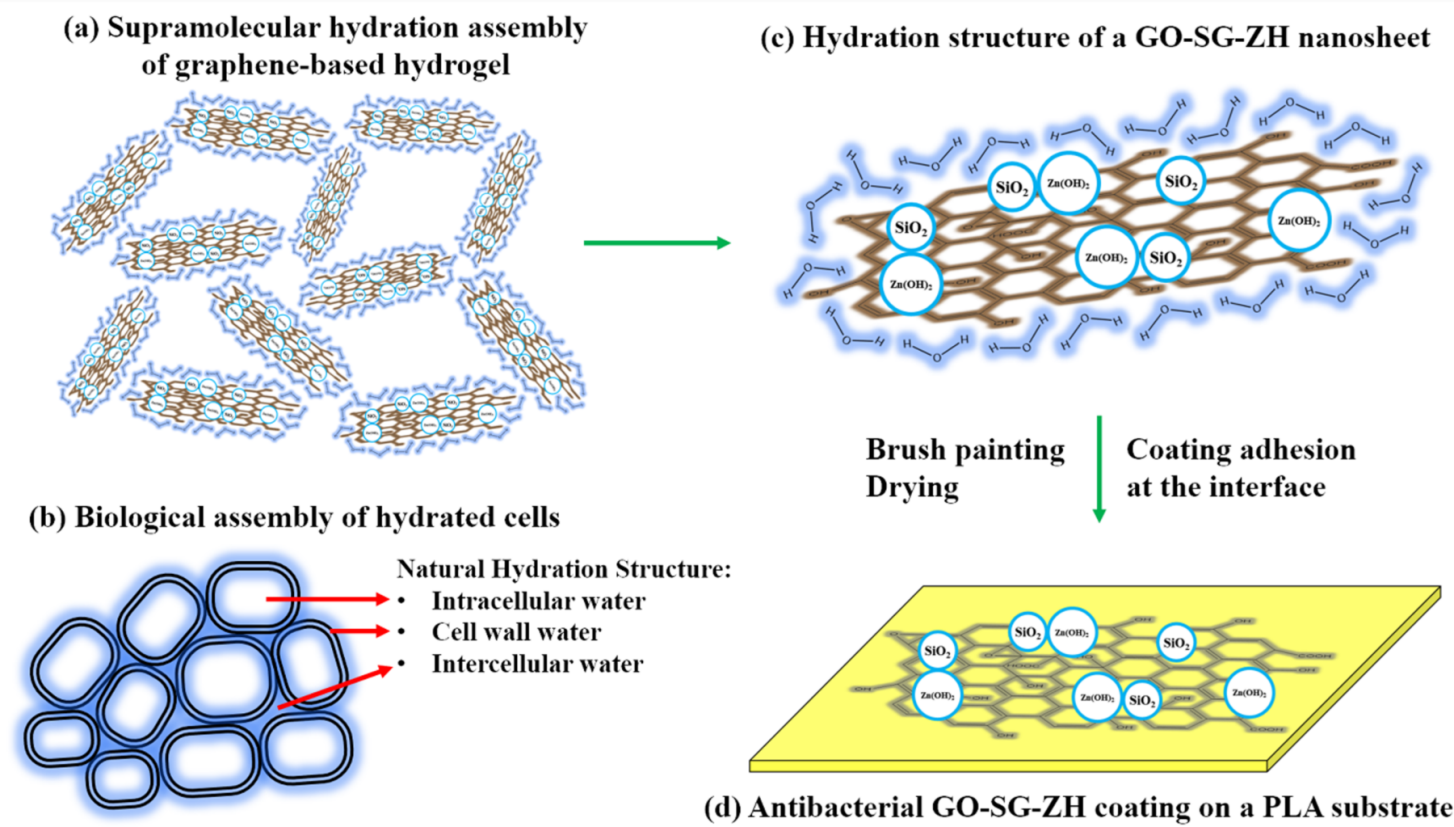
(a) Illustration of supramolecular self-assembly of a graphene-based hydrogel. (b) Depiction of a natural assembly of biological cells stabilized by hydration structures (water molecules on cell wall membranes are highlighted with blue color). (c) Drawing of a synthetic graphene-based nanosheet (GO-SG-ZH) covered by a hydration shell. (d) Presentation of a graphene-based coating (antibacterial GO-SG-ZH) that adheres to a substrate (PLA film).

The computational DFT results confirm the importance of supramolecular interaction of water intercalation in graphene-based structures. In this research, we synthesized graphene-based hydrogels of graphene oxide–nanosilica–zinc hydroxide nanocomposite (GO-SG-ZH hydrogel) as a supramolecular hydration structure. Figure 2a describes the supramolecular hydration assembly of the GO-SG-ZH hydrogel. GO nanosheets have brown color, and the hydration shells of water molecules is highlighted with blue color. Hydrophilic functional groups on GO nanosheets, SG nanoparticles, and ZH nanostructures are attractive to water molecules to form hydration shells on the surfaces. In addition to hydration layers, the three-dimensional assembly of graphene-based nanosheets provides high porosity as water reservoirs which supply water to intersheet spacings. High water content and large spacing distance in the hydrogel structure are key factors that prevent van der Waals and π–π interactions between graphene-based sheets.

Figure 2b depicts the hydrated assembly of biological cells in nature. The natural hydrated structure includes intracellular water, cell wall water, and intercellular water [32]. Hydration shells on the cellular walls or biomembranes are important to maintain cellular shape. The first bound water molecules on the biomembranes is a biointerfacial water layer (≈2.6 Å) which is responsible for primary hydration force [33–37]. Hydration forces in the range of 4–5 water layers contribute with repulsive energy to the biological system. Supramolecular hydrogen bonding between biostructures and water molecules leads to repulsive hydration forces when the surfaces are closely approached. The artificial structure of graphene-based hydrogel in Figure 2a is biomimetic to the natural system of biological cells described in Figure 2b. Hydration shells on GO-SG-ZH nanosheets, particularly the first interfacial water layer, generate hydration forces to maintain intersheet distances and nanoscale structures in the artificial system. The drawing in Figure 2c is the presentation of a graphene-based nanosheet with a first bound water layer which is responsible for the primary hydration force. In the next stage, after brush coating of the GO-SG-ZH hydrogel on a polylactide film, hydration shells are evaporated in the drying process, and the graphene-based nanosheets adhere to the substrate through electrostatic interaction, hydrogen bonding, and van der Waals interaction (Figure 2d).

**Figure 3:**
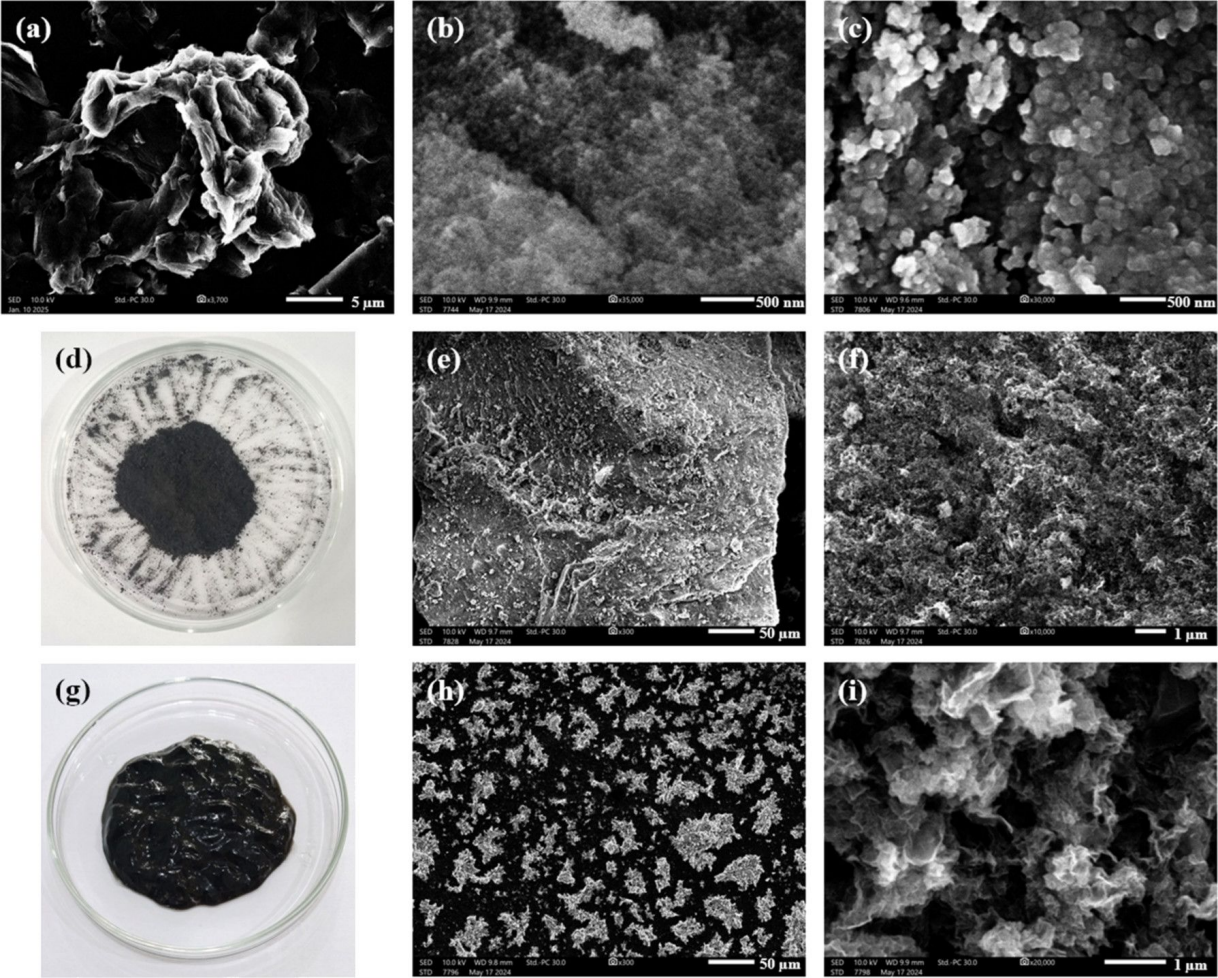
SEM images of GO nanosheets (a), SG nanoparticles (b), and SG-ZH nanoparticles (c). (d) Photograph of graphene oxide–nanosilica–zinc hydroxide powder. SEM images of particles and nanostructures in GO-SG-ZH powder with the scale bars of 50 µm (e) and 1 µm (f). (g) Photograph of hydrogel of graphene oxide–nanosilica–zinc hydroxide. SEM images of micro- and nanostructures in GO-SG-ZH hydrogel with the scale bars of 50 µm (h) and 1 µm (i).

Experimentally, GO nanosheets, SG, nanoparticles, and SG-ZH nanoparticles were synthesized and separately characterized as exhibited in SEM images in Figure 3a–c. Particularly, artificial nanocomposites of GO-SG-ZH powder (Figure 3d–f) and GO-SG-ZH hydrogel (Figure 3g–i) were prepared for comparative analysis. While the GO-SG-ZH powder is a dry solid (moisture ≈10%), the GO-SG-ZH hydrogel has a moisture content of 95% and viscoelastic behavior. The hydrogel was elastic to resist the deformation under gravitational force and also viscous to slowly deform (see Supporting Information File 1, Figure S2). The moisture content of the GO-SG-ZH hydrogel is comparable with those of natural cellular systems (moisture content of apple tissues is about 90%) [32]. In Figure 3e and 3f, SEM images of GO-SG-ZH powder show a macroscopic particle and its microscopic structure. Since the material was dehydrated, graphene-based nanostructures could be covalently cross-linked through esterification reaction of carboxyl and hydroxyl groups on GO nanosheets [38]. The GO-SG-ZH nanosheets agglomerated and stacked together to form big particles (size of hundreds of micrometers, Figure 3e). The GO-SG-ZH particles had low porosity or small spacing between graphene-based nanosheets (Figure 3f). Besides, the GO-SG-ZH hydrogel was spread on a carbon tape and dehydrated for SEM imaging (Figure 3h and 3i). Although GO-SG-ZH nanosheets agglomerated into microstructures (Figure 3h), the self-assembly of graphene-based nanosheets was different from the stacked morphology of the GO-SG-ZH powder. At a higher magnification of 20,000×, SEM image in Figure 3i revealed the porous structure with large spacing between graphene-based nanosheets. As a result, the GO-SG-ZH hydrogel is a three-dimensional assembly of water-intercalated graphene-based nanosheets.

**Figure 4:**
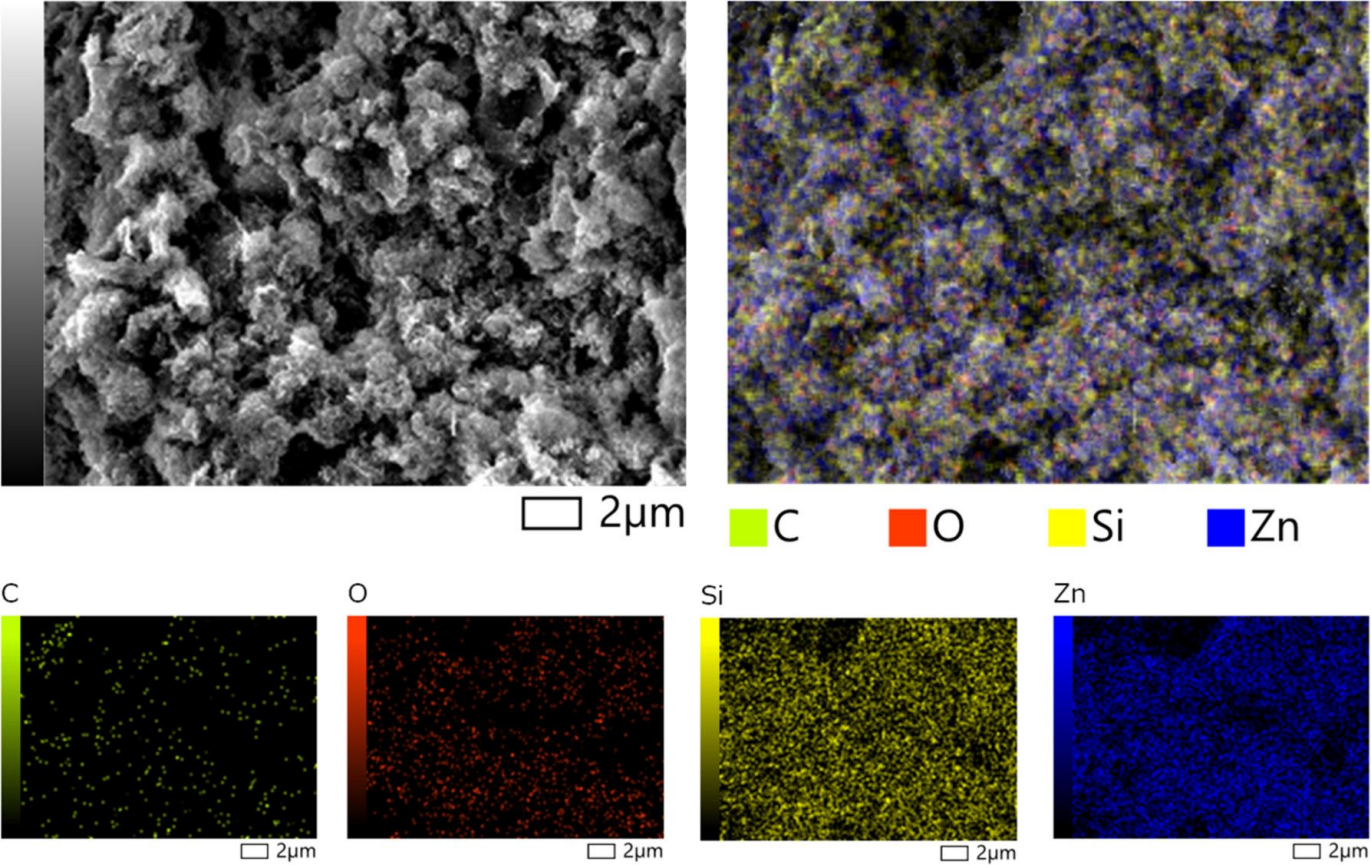
SEM-EDS elemental mapping of carbon atoms (green dots), oxygen atoms (red dots), silicon atoms (yellow dots), and zinc atoms (blue atoms) on the graphene-based nanostructure of GO-SG-ZH hydrogel after dehydration.

Results of EDS analysis in Table 2 disclose the elemental contents of the as-prepared GO-SG-ZH powder and hydrogel. Accordingly, atomic proportions of carbon, oxygen, silicon, and zinc elements in both nanocomposites are relatively similar. Theoretical contents of SiO_2_ and Zn(OH)_2_ in the GO-SG-ZH nanocomposite powder are estimated to be 37.92% and 33.12%, respectively, so the remaining content of GO nanosheets is about 28.96%. Similarly, SiO_2_, Zn(OH)_2_, and GO contents derived from the GO-SG-ZH hydrogel are calculated to be 34.89%, 38.43%, and 26.68%, respectively. In Figure 4, the elemental mapping of the three-dimensional structure of the GO-SG-ZH hydrogel showed the presence and distribution of carbon, oxygen, silicon, and zinc atoms on the graphene-based surfaces. SG-ZH nanoparticles and oxygen-containing functional groups on GO nanosheets are hydrophilic nanostructures which retain hydration layers on the graphene-based nanosheets, leading to the supramolecular hydration structure of the GO-SG-ZH hydrogel.

**Table 2:** EDS analysis of elemental compositions of GO nanosheets, SG nanoparticles, SG-ZH nanoparticles, GO-SG-ZH powder, and GO-SG-ZH hydrogel.

Materials	C (atom %)	O (atom %)	Si (atom %)	Zn (atom %)

GO	66.36 ± 2.13	33.64 ± 2.85	–	–
SG	–	76.44 ± 2.79	23.56 ± 1.36	–
SG-ZH	–	61.51 ± 4.68	22.31 ± 2.57	16.18 ± 1.57
GO-SG-ZH powder	18.78 ± 1.22	57.53 ± 2.03	12.64 ± 0.84	11.04 ± 0.58
GO-SG-ZH hydrogel	23.66 ± 1.59	51.90 ± 2.30	11.63 ± 0.95	12.81 ± 0.73

**Figure 5:**
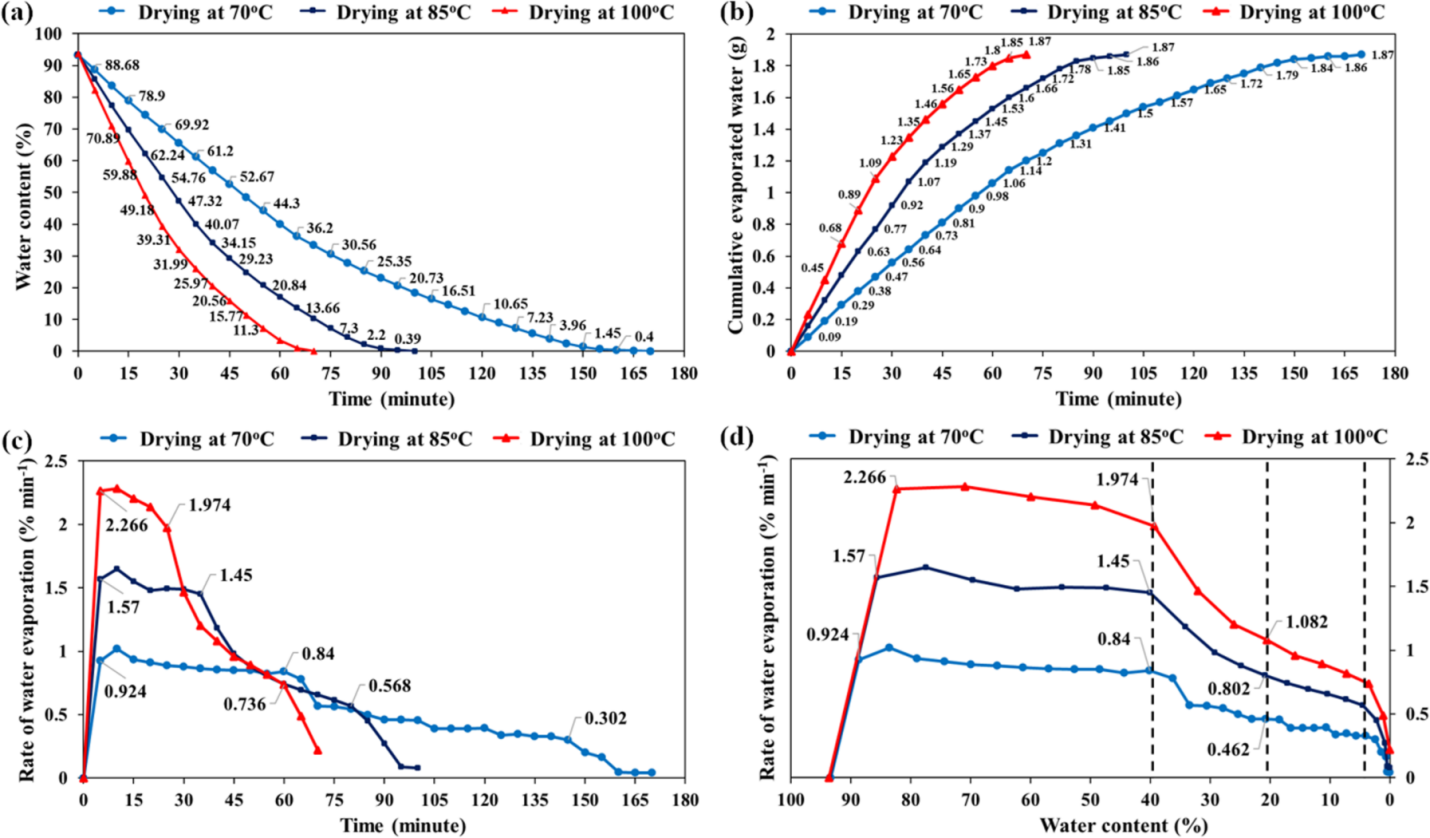
Analysis of moisture content and water evaporation of GO-SG-ZH hydrogel. (a) Curves of changing moistures of GO-SG-ZH hydrogel at different drying temperatures (70, 85, and 100 °C). (b) Curves of cumulative amount of evaporated water during drying at 70, 85, and 100 °C. (c) Plot of water evaporation rates with respect to drying time. (d) Plot of water evaporation rates with respect to water content in GO-SG-ZH hydrogel.

Water evaporation characteristics of the GO-SG-ZH hydrogel were recorded and analyzed during drying processes in the moisture analyzer (MX-50, resolution of 0.01%). Figure 5a describes the moisture curves during the drying process at 70, 85, and 100 °C, corresponding to the drying times of 170, 100, and 70 min, respectively. Although the corresponding drying times were different, the curves of cumulative evaporated water had a similar shape and amounted to 1.87 g (93.5% moisture, Figure 5b). The lower drying temperature of 70 °C was sufficient to evaporate the hydration layers in the hydrogel. A scientific report by Khan et al. demonstrated that the drying of apple tissues at 70 °C is critical to the rupturing of cell membranes, resulting in dehydration of the biological tissues [32]. In the drying process, water at the outer surface evaporated first, followed by the evaporation of intracellular water. Figure 5c and 5d exhibit the water evaporation rates as a function of drying time and water content in the GO-SG-ZH hydrogel. In the first period, the water evaporation rates increased to a plateau of constant drying rate, corresponding to the evaporation of free water molecules at the outer surface [39]. Then, the water evaporation curves entered into the decreasing rate period where there is deficiency of free water at the outer surface and water transport from the interior to the surface. The critical water content at which the first decreasing rate period began was identified to be about 40% (the left dashed line in Figure 5d). It is noticeable that at the water content of 20% (the middle dashed line in Figure 5d), the water evaporation rates at 70, 85, and 100 °C were approximately half the initial water evaporation rates at 5 min. There is a second decreasing rate period which occurred at the critical water content of 4% (the right dashed line in Figure 5d). Interestingly, in biological tissues, interfacial water integrated in cell walls is also about 2–5% [32]. It is explained that the significantly slower rates of water evaporation in the decreasing rate period are due to water movement from the interior to the surface and the hydrogen bonding of interfacial hydration layers on the hydrophilic nanostructures.

### Crystallography, functional group, aqueous dispersibility and hydration lubrication

**Figure 6:**
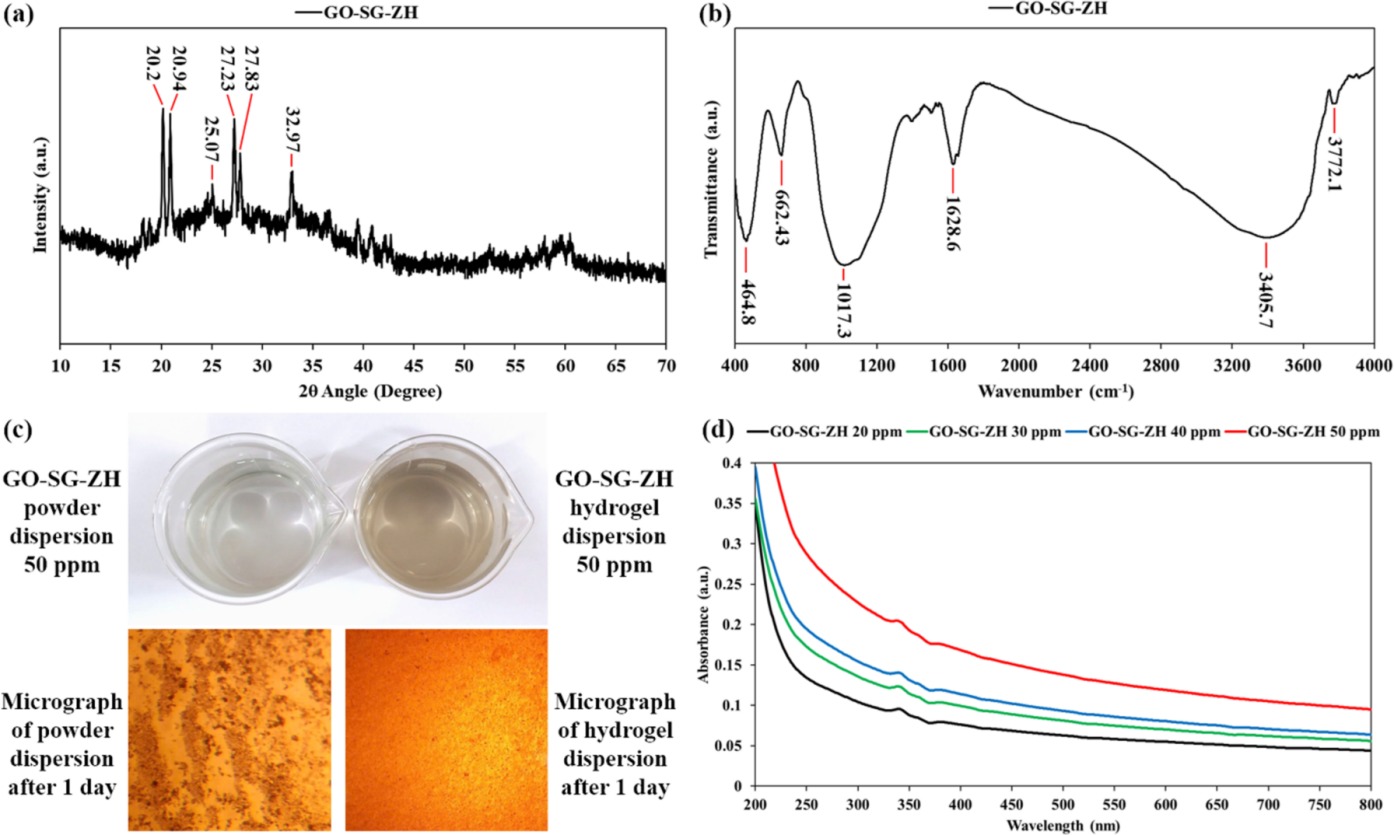
(a) XRD pattern of GO-SG-ZH powder. (b) FTIR spectrum of GO-SG-ZH powder. (c) Aqueous dispersions of GO-SG-ZH powder and hydrogel (concentrations of 50 ppm) and their sedimented particles after 1 day (visualized by the optical microscope). (d) UV–vis spectra of aqueous dispersions of GO-SG-ZH hydrogel.

Dry powder of the GO-SG-ZH nanocomposite was analyzed using XRD and FTIR. In Figure 6a, the XRD pattern exhibited sharp characteristic peaks of the Zn(OH)_2_ crystal at 2θ = 20.2°, 20.94°, 25.07°, 27.23°, 27.83°, and 32.97° [40–42]. Zinc hydroxide nanocrystals (ZH) were formed on the nanocomposite during the precipitation by alkaline ammonia in the synthesis process (see Methods section). The constituents of GO and SG nanomaterials had amorphous structures which did not give obvious peaks in the XRD pattern. Regarding the FTIR spectrum in Figure 6b, most of obvious peaks are attributed to functional groups of nanosilica. The vibration band at 3772.1 cm^−1^ is assigned to silanol groups on the nanosilica surface (Si–OH). The bands at 3405.7 and 1628.6 cm^−1^ are characteristic of stretching and bending modes, respectively, of water molecules adsorbed on the surface [43]. The bands at 1017.3 and 464.8 cm^−1^ represent the stretching vibrations of siloxane groups (Si–O–Si). Particularly, the FTIR band at 662.43 cm^−1^ is attributed to the bending vibration of Zn-O-Si bonds in the GO-SG-ZH nanocomposite [44].

Supramolecular systems with non-covalent interactions and reversible cross-links are recognized to provide extraordinary properties and applications [45–47]. Reversible self-assembly is an advantage of supramolecular graphene-based hydrogels in comparison with the powder form [13–16]. Hydration layers in between graphene-based sheets not only reduce intersheet binding energy (van der Waals force) but also generate repulsive forces for exfoliating the macroscopic assembly into nanoscale structures especially under external sonication and mechanical stimuli. Reversible self-assembly of graphene-based nanosheets in water is essential to many applications, such as adsorption, photocatalysis, biosensing, drug delivery, aqueous paints, and multifunctional coatings [48–50]. Figure 6c exhibits the aqueous dispersions derived from the sonication of GO-SG-ZH hydrogel and powder in water (see Supporting Information File 1, Figure S3). Ultrasound waves vibrated water molecules and created cavitation in the hydrogel structure, leading to the exfoliation of graphene-based nanosheets in water. It is notable that low concentrations (≤50 ppm) are necessary to obtain homogenous dispersions. The ultrasonic dispersion of GO-SG-ZH hydrogels was faster and clearer due to the high content of water intercalation. The GO-SG-ZH powder contained about 10% of water and approximate 60% of nanosilica and zinc hydroxide nanoparticles, which functioned as spacing layers between graphene-based nanosheets. Therefore, the ultrasonic treatment of GO-SG-ZH powder in water also yielded a homogeneous dispersion. However, the aqueous dispersion of GO-SG-ZH powder was not completely exfoliated in the aqueous environment and quickly settled down. Stacked agglomerates of GO-SG-ZH powder at the bottom of the dispersion were observed in the micrograph of Figure 6c, while the micrograph of the dispersion of GO-SG-ZH hydrogel showed the absence of stacked structures. In Figure 6d, UV–vis spectra of aqueous dispersions of GO-SG-ZH hydrogel present light absorption in the ultraviolet range (200–400 nm) that was proportional to the colloidal concentrations (50, 40, 30, and 20 ppm). Small absorption peaks at 340 and 360 nm correspond to nanosilica and zinc hydroxide nanoparticles, respectively [21,43]. In addition, hydration lubrication in supramolecular graphene-based hydrogels is important to applications in paints and coatings [51–52]. While the GO-SG-ZH powder could not be directly used for brush painting, the GO-SG-ZH hydrogel can be easily coated on various substrates using a simple brush. In the scientific literature, it is elucidated that water layers between graphene-based nanosheets significantly lower the interfacial frictions of the nanomaterials [53–55]. In this study, hydration lubrication makes supramolecular graphene-based hydrogels suitable for direct brush coating on PLA films.

### Light transmittance spectroscopy, microscopic structure and elemental composition of graphene oxide–nanosilica–zinc hydroxide coating on polylactide films

**Figure 7:**
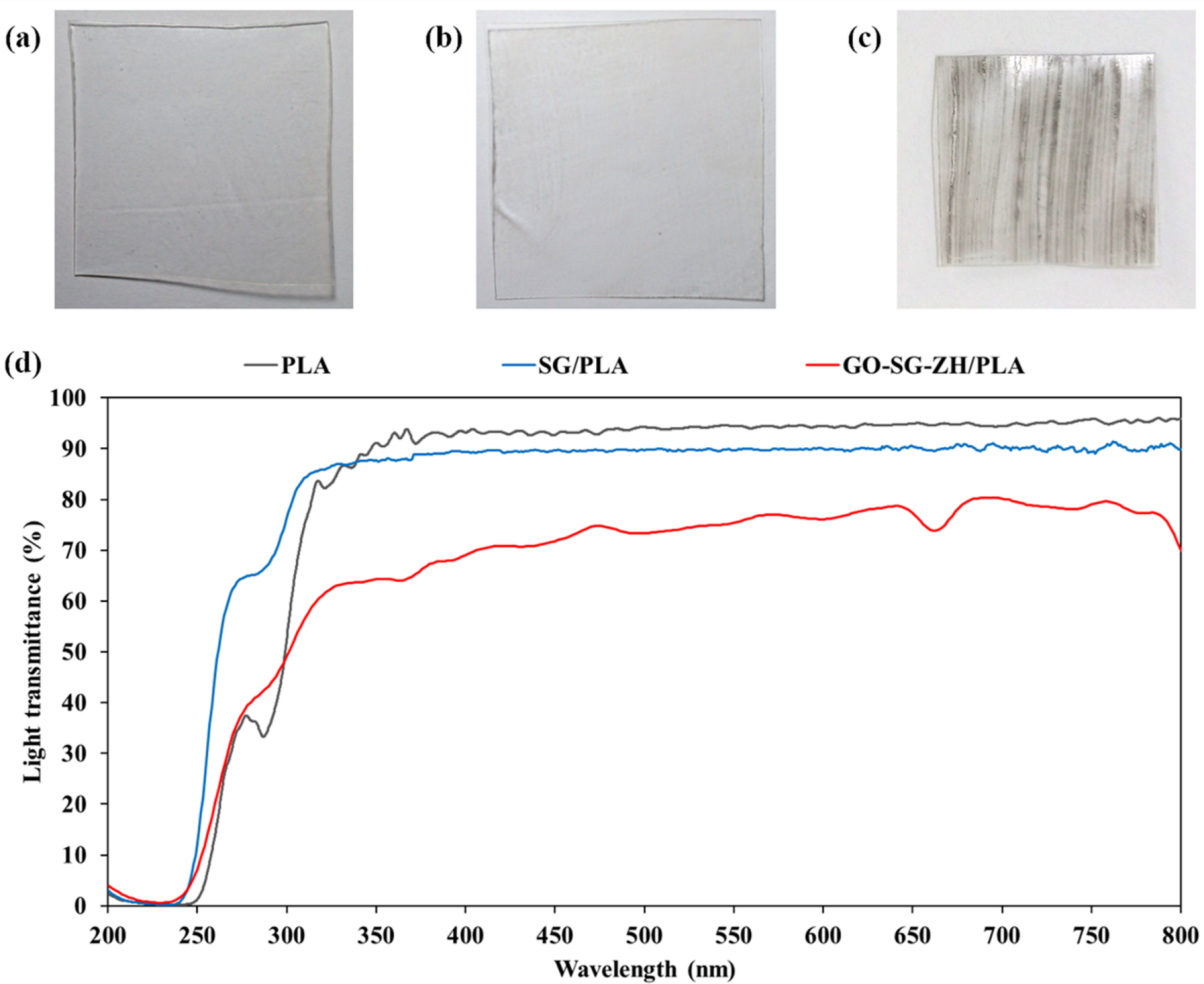
(a) A blank polylactide film (PLA). (b) A polylactide film coated with nanosilica (SG/PLA). (c) A polylactide film coated with graphene oxide–nanosilica–zinc hydroxide (GO-SG-ZH/PLA). (d) Light transmittance spectroscopy of thin films of PLA, SG/PLA, and GO-SG-ZH/PLA.

Nanosilica hydrogels and graphene oxide–nanosilica–zinc hydroxide hydrogels were utilized as aqueous paints for brush coating on PLA films. After drying, thin coatings of SG and GO-SG-PLA were formed on the plastic substrates. Regarding appearance, while the blank PLA film was clearly transparent (Figure 7a), the SG/PLA film was slightly opaque (Figure 7b), and the GO-SG-ZH/PLA film was stripy with black lines of GO color (Figure 7c). Light transmittance spectra in Figure 7d show the transparency levels of the plastic films. In the visible light range of 400–700 nm, the average light transmittance values of blank PLA, SG/PLA, and GO-SG-ZH/PLA films are 94%, 90%, and 75% respectively. The SG coating made the transparency decrease 4%, and the GO-SG-ZH coating resulted in the transparency decline of 19% due to the white color of ZH nanoparticles and black color of GO nanosheets.

**Figure 8:**
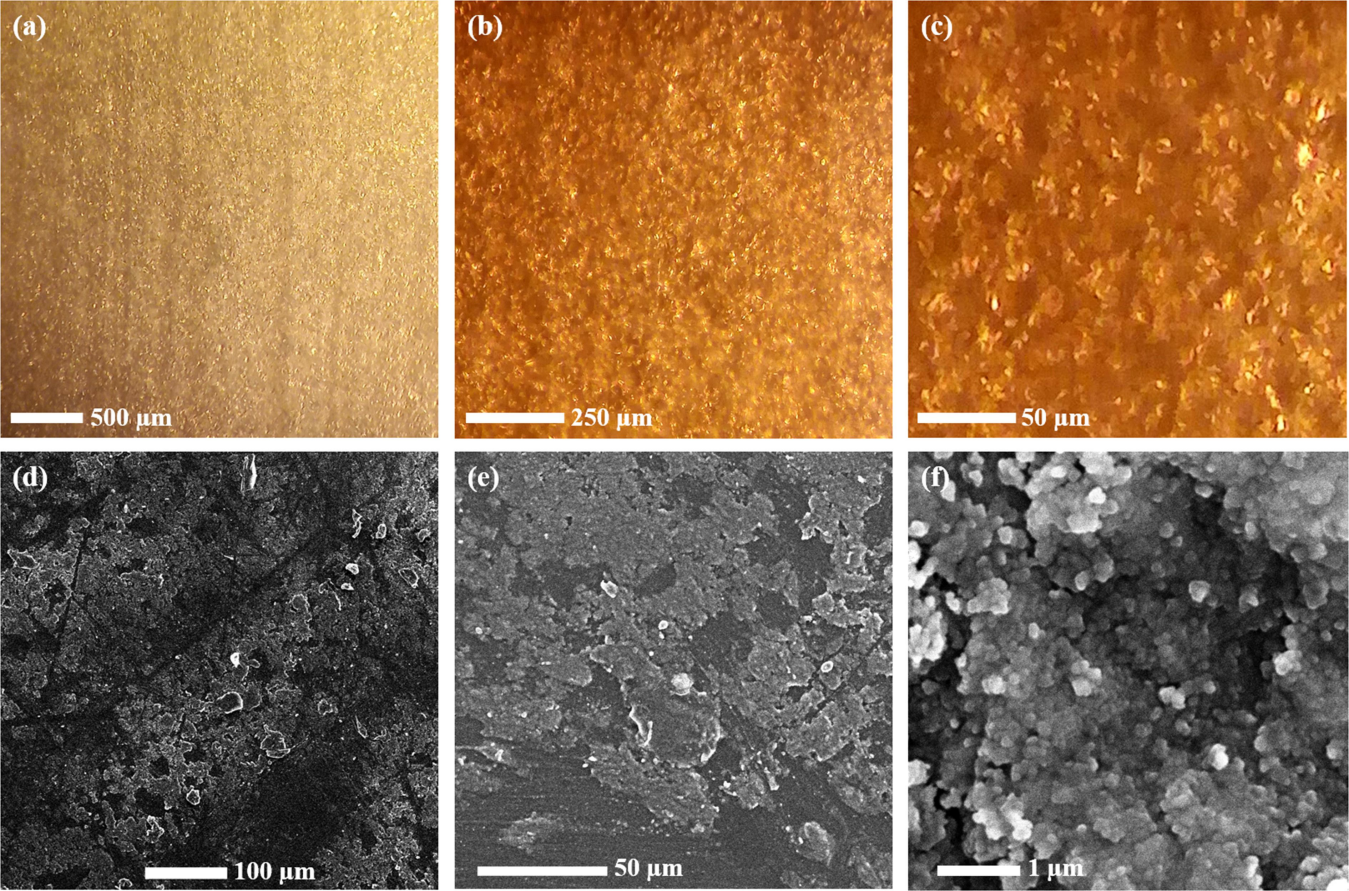
(a, b, c) Micrographs of morphology and structure of GO-SG-ZH coating on a PLA film. (d, e, f) SEM images of GO-SG-ZH coating on a PLA film.

Microscopic structures of the GO-SG-ZH coating on a PLA film were observed and imaged using the optical stereo microscope. Since the PLA substrate was almost transparent, pictures in Figure 8a–c showed GO nanosheets, SG nanoparticles, and ZH nanostructures of the GO-SG-ZH coating. Reflected light from the GO-SG-ZH coating gave a three-dimensional vision of the coating texture. The nanostructures in the coating morphology are well-distributed. In Figure 8c, two-dimensional shapes of GO nanosheets are visualized. In Figure 8d–f, SEM provided high-resolution images of the GO-SG-ZH/PLA film. The brush-coated layer of the GO-SG-ZH nanocomposite was not completely uniform since rough coating morphology was observed on the substrate surface. Two-dimensional graphene-based sheets appeared in Figure 8e, and nanoparticles of SG and ZH were shown in Figure 8f. Integrated EDS analysis presented the elemental composition on the surface of the GO-SG-ZH/PLA film (Table 3). With the atomic contents of 8.13% of silicon and 6.36% of zinc, the atomic proportions of SiO_2_ and Zn(OH)_2_ in the nanocomposite were estimated to be 24.39% and 19.08%, respectively.

**Table 3:** EDS elemental composition of the GO-SG-ZH coating on the PLA substrate.

Materials	C (atom %)	O (atom %)	Si (atom %)	Zn (atom %)

SG-GO-ZH/PLA film	45.86 ± 1.84	39.64 ± 1.98	8.13 ± 0.70	6.36 ± 0.47

### Antibacterial properties of graphene oxide–nanosilica–zinc hydroxide in hydrogel and in coating structures

**Figure 9:**
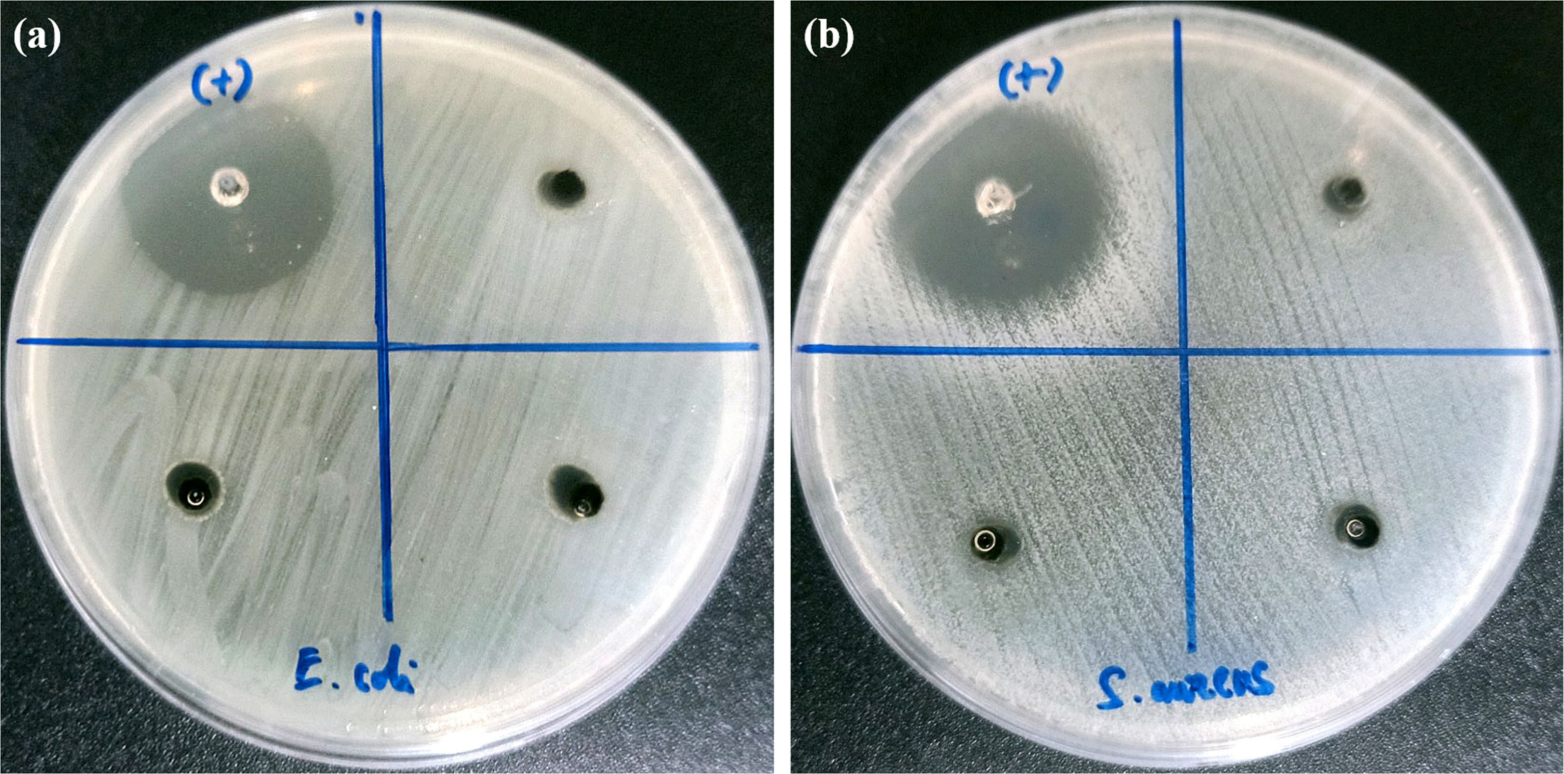
Agar well diffusion assay of the GO-SG-ZH hydrogel presents antibacterial activities of the GO-SG-ZH hydrogel against *E. coli* (a) and *S. aureus* (b).

The EDS analysis in Table 2 showed that the solid nanocomposite of GO-SG-ZH hydrogel was composed of 12.81% zinc atoms (derived from the Zn(OH)_2_ constituent). ZH nanoparticles and GO nanosheets in the GO-SG-ZH hydrogel are antibacterial and antibiofilm agents with low toxicity for food packaging and biomedical applications [56–57]. The main antibacterial mechanism of GO nanosheets is cell membrane damage caused by direct contact of GO sharp edges with bacterial membranes [57–58]. The crucial antibacterial activity of ZH nanostructures is the delivery of Zn^2+^ ions to disrupt bacterial membranes and intracellular processes [59–60]. Antibacterial activity of the GO-SG-ZH hydrogel was tested in agar well diffusion assays (Figure 9). The photographic results showed inhibition zones against *E. coli* (Figure 9a) and *S. aureus* (Figure 9b). The inhibition zones resulted from the diffusion of ZH nanoparticles and Zn^2+^ cations from the hydrogel to the surrounding agar. As the GO-SG-ZH hydrogel is antibacterial, the brush coating of the GO-SG-ZH hydrogel on PLA films produced an antibacterial coating on the substrate.

**Figure 10:**
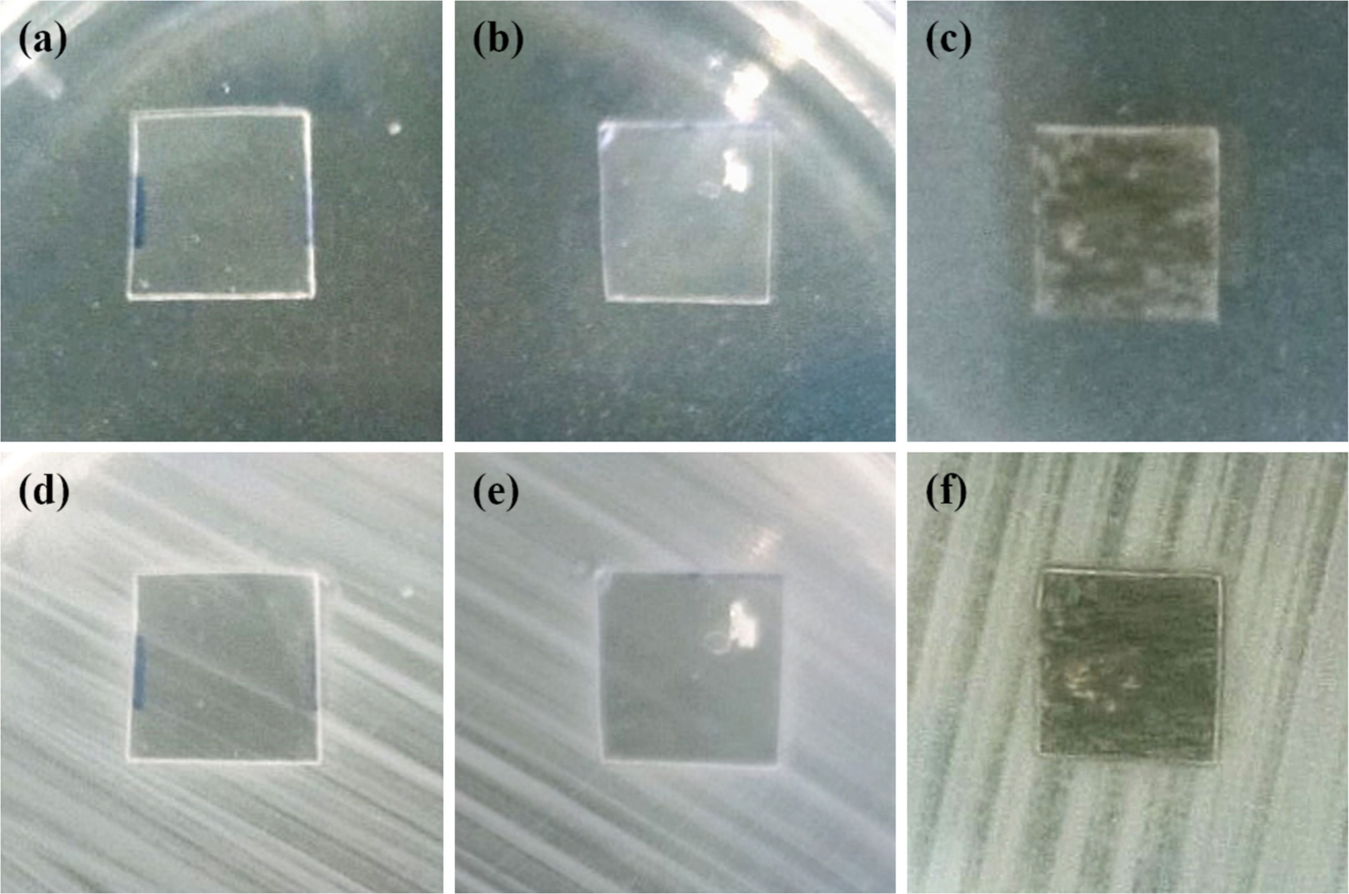
Antibacterial test of uncoated and coated PLA films against the growth of *E. coli* biofilm. (a, b, c) Pictures of a PLA film (a), an SG/PLA film (b), and a GO-SG-ZH/PLA film (c) on agar plates inoculated with *E. coli* bacteria (before incubation). (d, e, f) Pictures of the PLA film (d), SG/PLA film (e), and GO-SG-ZH/PLA film (f) on the agar/biofilm plates after incubation at 37 °C for 24 h.

Antibacterial tests of uncoated and coated PLA films are described in Figure 10, where the interfaces between PLA films and agar/*E. coli* plates are displayed. Before the incubation process, *E. coli* bacteria did not grow to biofilms (Figure 10a–c). After incubation at 37 °C for 24 h, stripy biofilms of *E. coli* bacteria were formed on the agar plates (Figure 10d–f). While stripy patterns were observed in the areas of blank PLA film (Figure 10d) and SG/PLA film (Figure 10e), the GO-SG-ZH/PLA film presented an inhibition zone at the vicinity of its boundary (Figure 10f). PLA and SG/PLA were not antibacterial materials, and the GO-SG-ZH coating was effective against the growth of *E. coli* biofilm on the coating surface. The antibiofilm result is attributed to the antibacterial activities of GO nanosheets and ZH nanoparticles. Regarding the antibacterial mechanism of the nanocomposite coating, direct contact of bacterial cells with sharp nanostructures of the coating is the cause of membrane damage and cell inactivation. Zn^2+^ cations released from ZH nanoparticles and reactive oxygen species generated by ZH nanoparticles and GO nanosheets are effective bactericidal agents that disrupt bacterial cells [56–60]. The antibacterial actions and results are meaningful for preventions of biofilm formation and surface-mediated infections [14,25,61]. GO-SG-ZH/PLA is a good material for packaging and biomedical applications thanks to its antibiofilm, safety, and biodegradability properties.

### Stability of graphene oxide–nanosilica–zinc hydroxide coatings on polylactide films in aqueous environments

**Figure 11:**
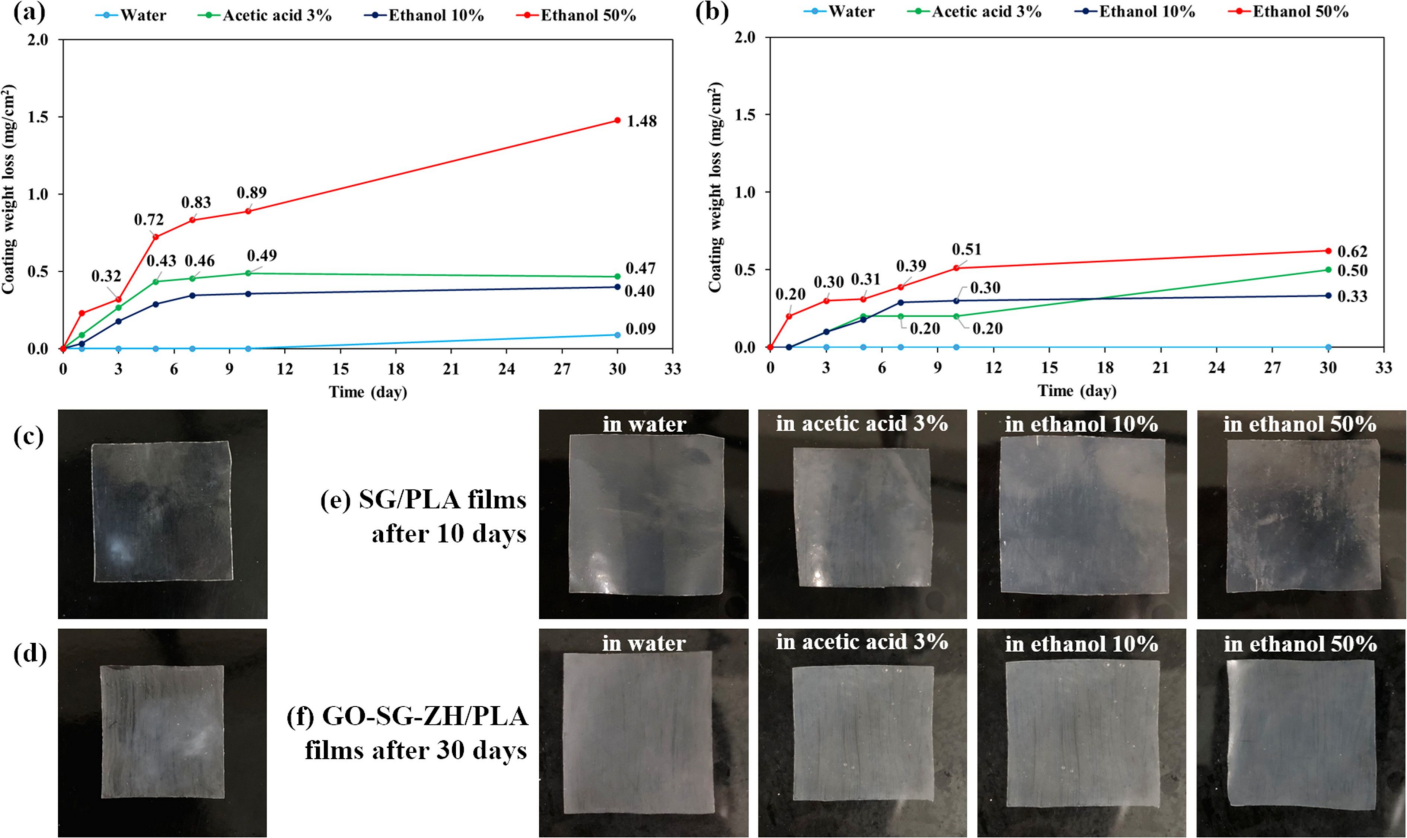
Stability testing of SG/PLA and GO-SG-ZH/PLA films in an environment simulating aqueous food (water, 3% acetic acid, 10% ethanol, and 50% ethanol). (a, b) Graphs of weight losses of SG coating (a) and GO-SG-ZH coating (b) in the period of 30 days in aqueous solutions. (c, d) Initial SG/PLA film (c) and GO-SG-ZH/PLA film (d). (e, f) Pictures of SG/PLA films after 10 days (e) and GO-SG-ZH/PLA films after 30 days (f) in aqueous environments.

SG/PLA and GO-SG-ZH/PLA films were immersed in an environment simulating aqueous food for one month. The stability of the coatings in aqueous environments was measured by calculating the loss of coating weight. These experimental tests are useful for studying packaging materials and chemical releases over a time period in environments simulating food [14,27]. Line charts in Figure 11 report the weight losses of SG (Figure 10a) and GO-SG-ZH/PLA (Figure 10b) coatings after 1, 3, 5, 7, 10 and 30 days in aqueous solutions containing water, 3% acetic acid, 10% ethanol, and 50% ethanol. Both coatings were quite stable in pure water as the weight losses were insignificant even after 30 days. However, acidic and alcoholic solutions gave more notable effects on coating stability. Especially, the weight losses of SG coating in 50% ethanol were 0.32 mg/cm^2^ after 3 days and 1.48 mg/cm^2^ after 30 days. The high coating weight loss indicated that the coating of silica nanoparticles was not stable in 50% ethanol solution (equivalent to a fatty food environment). Besides, the coating of GO-SG-ZH nanocomposite showed weight losses of 0.3 mg/cm^2^ after 3 days and 0.62 mg/cm^2^ after 30 days in 50% ethanol. Although the coating weight losses were considerable in the first 3 days, the trend curve became steady in the period from 3 to 30 days. The GO-SG-ZH coating was more stable than the SG coating on PLA films. As demonstrated in our previous paper, the coating of zinc hydroxide nanoparticles was not stable in aqueous solutions [14]. In this study, the stability of GO-SG-ZH coating is attributed to the role of graphene-based nanosheets. Large GO nanosheets are important to provide effective surface area for coating adhesion to the flat substrate and cross-linking in the coating network. Interactions at the interface between the coating and substrate include electrostatic interaction, hydrogen bonding, and van der Waals attraction. In the molecular dynamics simulations by Hasheminejad et al., the interfacial interaction energy between graphene oxide nanosheet and polylactide matrix is assigned to van der Waals forces and hydrogen bonds [62]. The bonding network of GO-SG-ZH nanosheets in the coating is another reason for the coating stability in environments simulating aqueous food.

### Mechanical properties of polylactide films with nanosilica-based and graphene-based coatings

**Figure 12:**
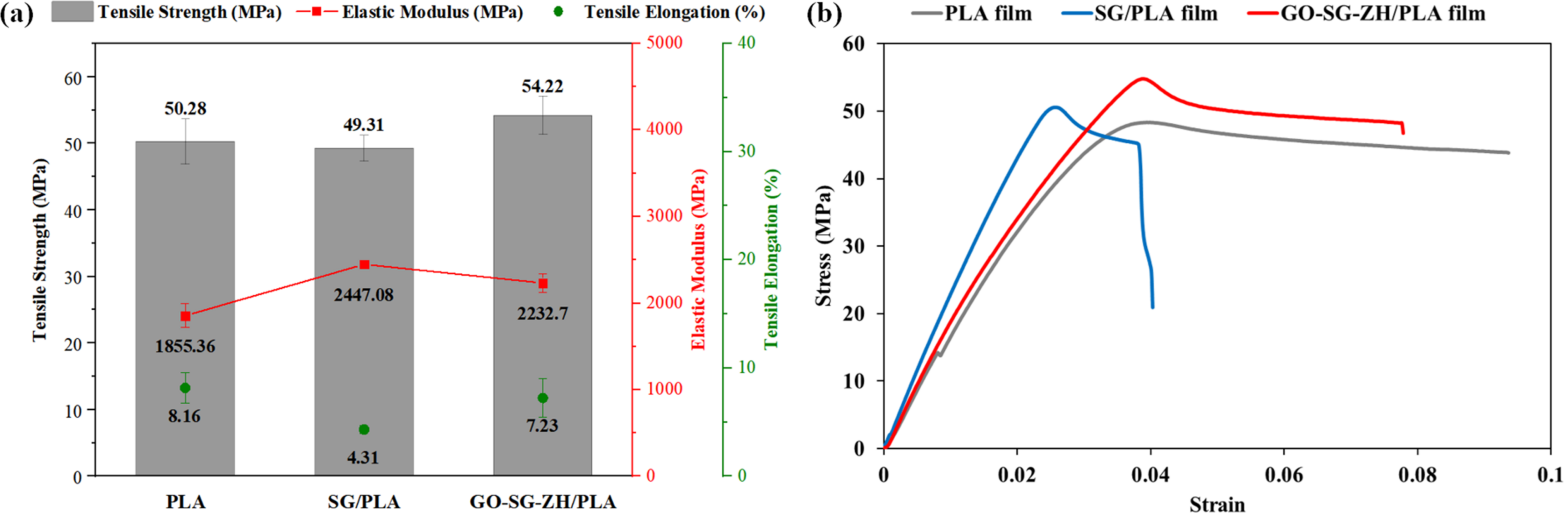
(a) Graph of tensile strength, elastic modulus, and tensile elongation. (b) Typical stress–strain curves of PLA, SG/PLA, and GO-SG-ZH/PLA films.

The thin coatings of SG and GO-SG-ZH considerably affected the mechanical properties of plastic films. Tensile testing results of blank PLA, SG/PLA, and GO-SG-ZH/PLA films are described in Figure 12 and summarized in Table 4. Additional data of measurement values and stress–strain curves are given in Supporting Information File 1, Table S1, Figure S4, Table S2, and Figure S5. Our previous paper presented that GO-ZnO coating on PLA film led to an increase of elastic modulus and a decrease of tensile elongation [14]. Similar trends were also noted in the tensile properties of coated PLA films in this study. Elastic moduli of SG/PLA and GO-SG-ZH/PLA films rose to 2447.08 ± 27.71 MPa and 2232.7 ± 105.52 MPa, which were respectively 31.89% and 20.34% higher than that of blank PLA film. Nanosilica and graphene-based nanosheets were nanostructures with high elastic modulus for reinforcement of PLA films through load transfer mechanism. High elasticity of SG and GO-SG-ZH coatings led to the increases in elastic moduli of the coated films. Besides, considerable decrease of elongation was observed due to the propagation of cracks from the coatings to the substrate. The tensile strength of the GO-SG-ZH/PLA film (54.22 ± 2.86 MPa) was slightly higher than that of the blank PLA film. The enhancement of tensile strength of the GO-SG-ZH/PLA film is explained due to the load transfer from the polylactide substrate to the graphene-based coating during the tensile process. Effective coating adhesion to the substrate and high elastic modulus of graphene-based nanosheets contributed to the higher tensile properties. Supporting Information File 1, Figure S6 shows SEM images of surfaces at the fracture of a GO-SG-ZH/PLA film generated by the tensile measurement.

**Table 4:** Mechanical properties of blank PLA, SG/PLA, and GO-SG-ZH/PLA thin films.

Materials	Tensile strength (MPa)	Elastic modulus (MPa)	Tensile elongation (%)

PLA	50.28 ± 3.4	1855.36 ± 138.56	8.16 ± 1.43
SG/PLA	49.31 ± 1.93	2447.08 ± 27.71	4.31 ± 0.36
GO-SG-ZH/PLA	54.22 ± 2.86	2232.7 ± 105.52	7.23 ± 1.77

## Conclusion

Supramolecular graphene-based hydrogels are bioinspired structures which are biomimetic to natural hydration structures of cellular membranes, proteins, and other biomolecules. While hydration shells participate in the shaping and dynamics of biological structures, water intercalation in graphene-based hydrogels is proposed to reduce intersheet van der Waals interaction, generate repulsive hydration forces, and facilitate hydration lubrication of graphene-based nanosheets. DFT calculations showed that a water layer in AB bilayer graphene enlarges the intersheet distance from 3.459 to 6.626 Å, and consequently leads to a reduction of 37.5% in intersheet binding energy of the van der Waals force. In our experiments, sustainable green chemistry approaches are used to synthesize graphene oxide, silica gel, nanosilica–zinc hydroxide, and graphene oxide–nanosilica–zinc hydroxide nanocomposites. The chemical methods used saved chemical reagents and production energy, converted rice hush ash waste into nanosilica, improved materials quality, and contributed to environmental sustainability. The GO-SG-ZH hydrogel is a supramolecular hydration structure with the advantages of aqueous dispersibility, antibacterial activity, and hydration lubrication. Water evaporation analysis suggested that the last 4% of water in the GO-SG-ZH hydrogel are interfacial hydration shells on graphene-based nanosheets. The first water shell is crucially responsible for primary hydration forces between nanostructures. Additionally, hydration lubrication is another interesting effect of water-intercalated graphene-based systems. As graphene-based nanosheets in the hydrogel structure are in non-stacking state, they can slide on each other owing to water lubrication and low interfacial friction. After brush coating and water evaporation, the graphene-based coating is adhered to the polylactide substrate through interfacial interactions. The GO-SG-ZH/PLA films showed antibacterial activity, coating stability, and enhanced tensile properties. In summary, the supramolecular hydration structure of graphene-based hydrogels is a prospective nanotechnology approach to advance nanoscale structures and interfaces for a variety of applications.

## Supporting Information

Figure of SEM-EDS analyses of graphene-based powder and hydrogel, figures of nanocomposite hydrogel and its dispersions in water, figures and tables of tensile testing of coated polylactide films, and figure of SEM images of fractured polylactide nanocomposite films.

File 1 Additional figures and tables.

## Data Availability

Data generated and analyzed during this study is available from the corresponding author upon reasonable request.
