# Supplementary material for: Supramolecular hydration structure of graphene-based hydrogels: density functional theory, green chemistry and interface application
Source: Beilstein J Nanotechnol. 2025 Jun 4;16:806–22. doi: 10.3762/bjnano.16.61 (PMC12152314; doi:10.3762/bjnano.16.61)
Supplement: File 1 — Additional figures and tables. [file Beilstein_J_Nanotechnol-16-806-s001.pdf]

BEILSTEIN JOURNAL OF NANOTECHNOLOGY

Supporting Information

for

Supramolecular hydration structure of graphene-based hydrogels: density functional theory, green chemistry and interface application

Hon Nhien Le, Duy Khanh Nguyen, Minh Triet Dang, Huyen Trinh Nguyen,
Thi Bang Tam Dao, Trung Do Nguyen, Chi Nhan Ha Thuc and Van Hieu Le

Beilstein J. Nanotechnol. **2025,** *16,* 806–822. doi:10.3762/bjnano.16.61

Additional figures and tables

License and Terms: This is a supporting information file under the terms of the Creative Commons Attribution License (https://creativecommons.org/licenses/by/4.0). Please note that the reuse, redistribution and reproduction in particular requires that the author(s) and source are credited and that individual graphics may be subject to special legal provisions.
The license is subject to the *Beilstein Journal of Nanotechnology* terms and conditions: (https://www.beilstein-journals.org/bjnano/terms)

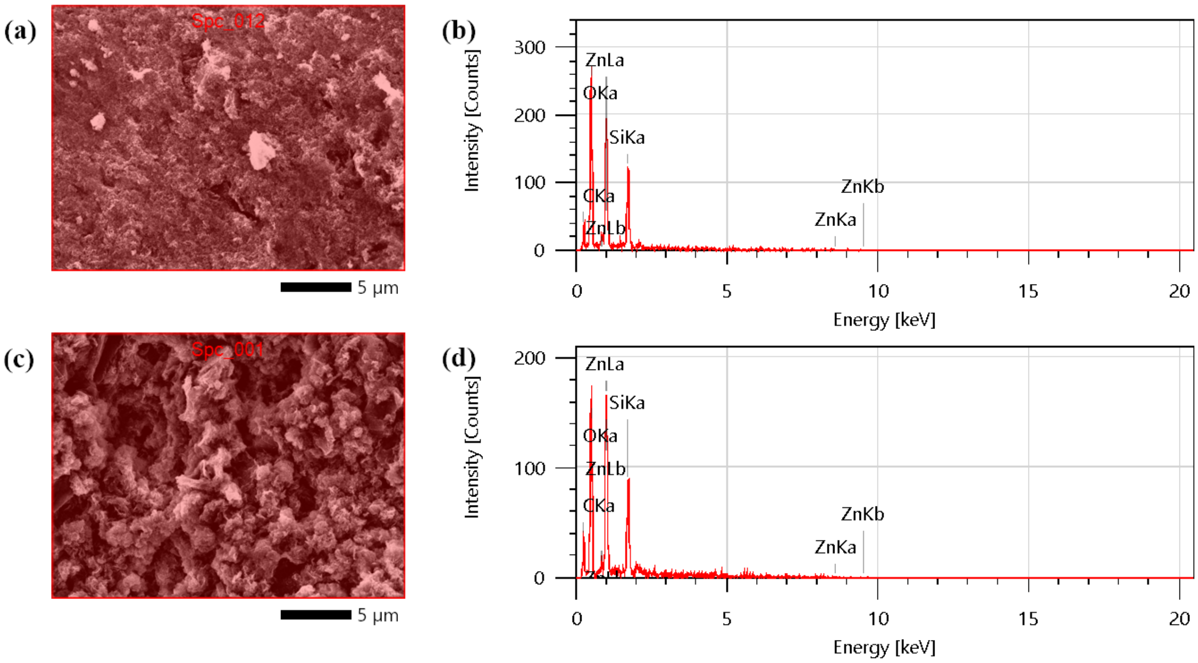

Figure S1: SEM–EDS analysis. SEM image (a) and EDS spectrum (b) of the analyzed area in GO-SG-ZH powder. SEM image (c) and EDS spectrum (d) of the analyzed area in GO-SG-ZH hydrogel.

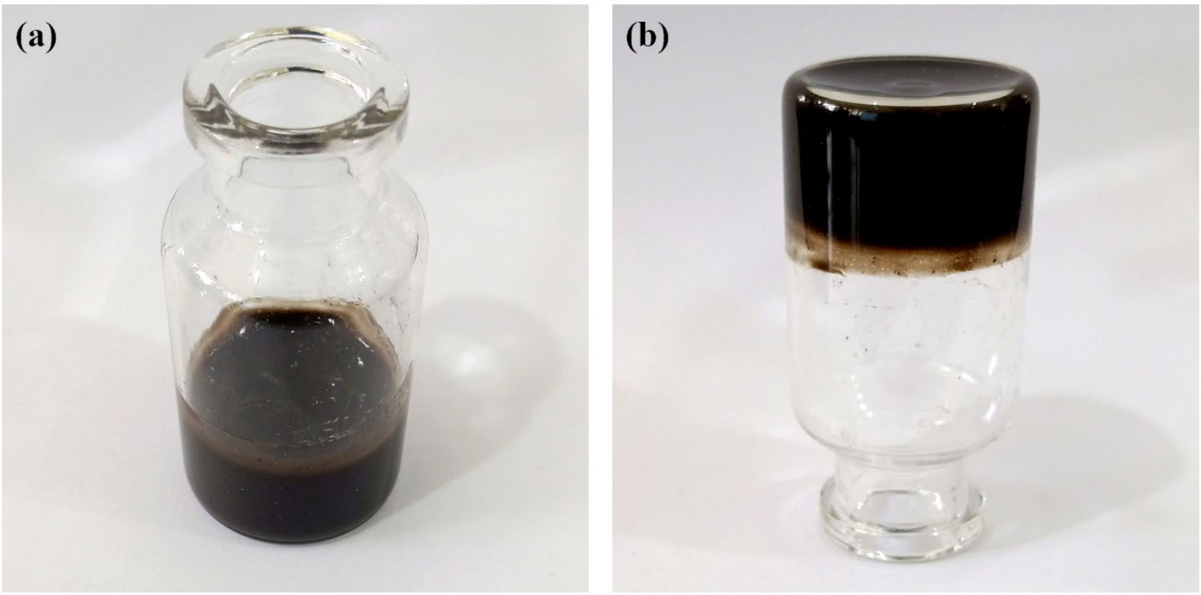

Figure S2: Photographs of GO-SG-ZH hydrogel.

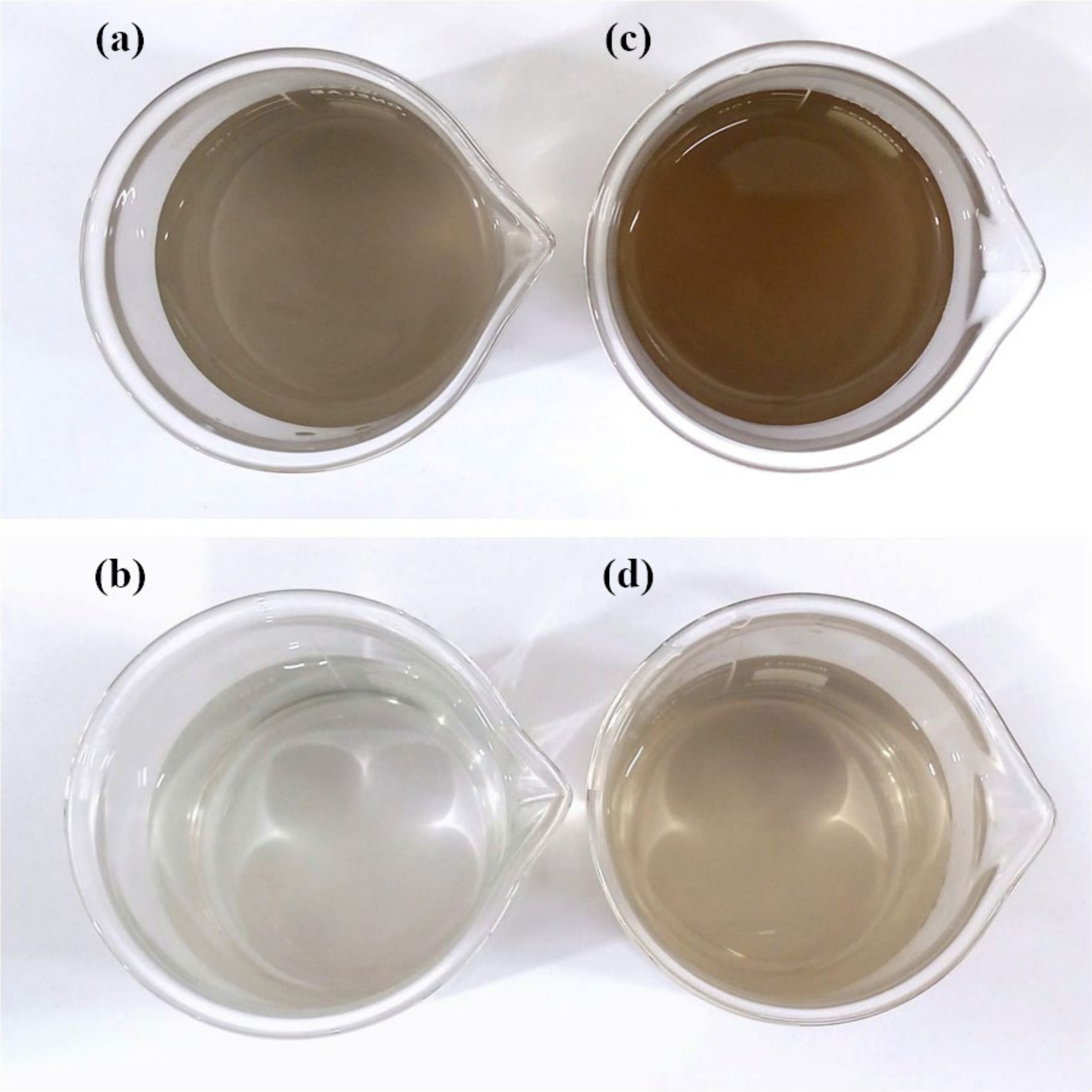

Figure S3: Aqueous dispersions of GO-SG-ZH powder in water at the concentrations of 200 (a) and 50 ppm (b). Aqueous dispersions of GO-SG-ZH hydrogel at the concentrations of 200 (c) and 50 ppm (d).

Table S1: Value results of tensile measurement of SG/PLA films.

No.	Maximum Load N	Elasticity Modulus MPa	Max Load Stress MPa	Max Load Strain strain	Max Load Elong %	Break Elong %	Break Energy kgf-mm	Sample Thickness mm
1	58.35	2426.64	51.18	0.03	2.68	4.82	4.14	0.190
2	59.10	2457.00	50.51	0.03	2.54	4.00	3.25	0.195
3	50.65	2481.77	46.90	0.02	2.36	4.16	2.96	0.180
4	46.70	2422.90	48.65	0.02	2.44	4.27	2.86	0.160
Average	53.70	2447.08	49.31	0.03	2.50	4.31	3.30	0.181
Std	6.03	27.71	1.93	0.01	0.14	0.36	0.58	0.015

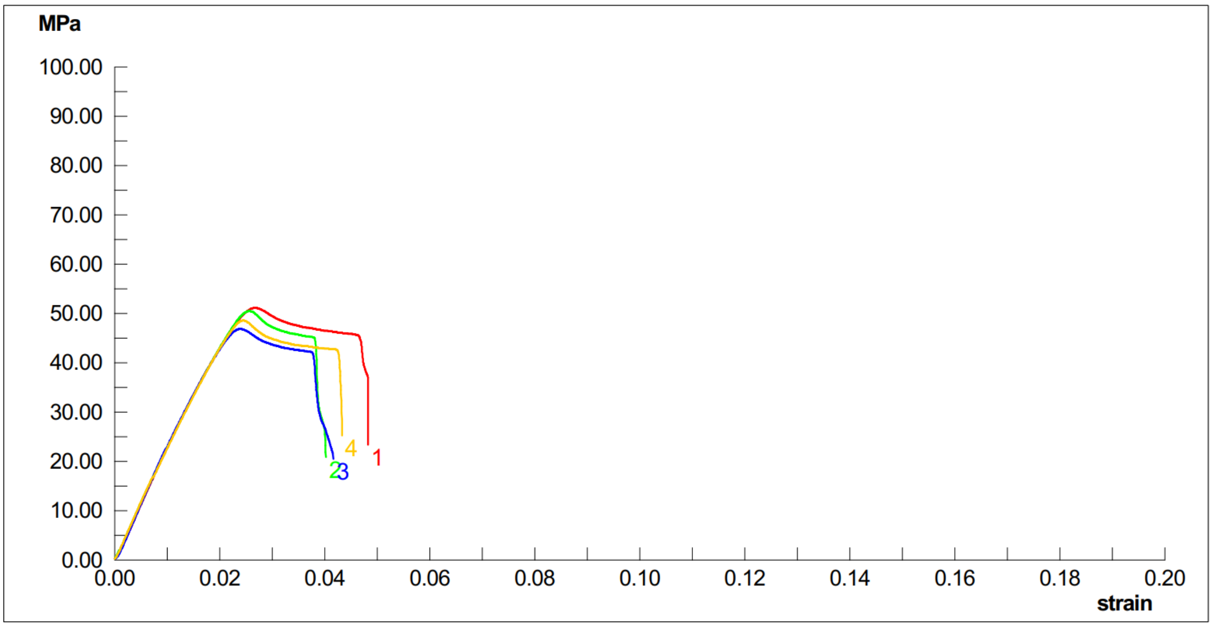

Figure S4: Stress–strain curves of tensile testing of SG/PLA films.

Table S2: Value results of tensile measurement of GO-SG-ZH/PLA films.

No.	Maximum Load N	Elasticity Modulus MPa	Max Load Stress MPa	Max Load Strain strain	Max Load Elong %	Break Elong %	Break Energy kgf-mm	Sample Thickness mm
1	62.40	2122.40	54.74	0.04	3.86	7.76	7.33	0.190
2	68.65	2118.82	57.21	0.04	4.42	4.57	3.52	0.200
3	63.65	2314.17	55.83	0.04	4.03	9.13	9.08	0.190
4	56.65	2268.11	49.69	0.03	3.32	6.45	5.54	0.190
5	61.15	2339.98	53.64	0.04	3.59	8.24	7.58	0.190
Average	62.50	2232.70	54.22	0.04	3.84	7.23	6.61	0.192
Std	4.34	105.52	2.86	0.00	0.42	1.77	2.14	0.004

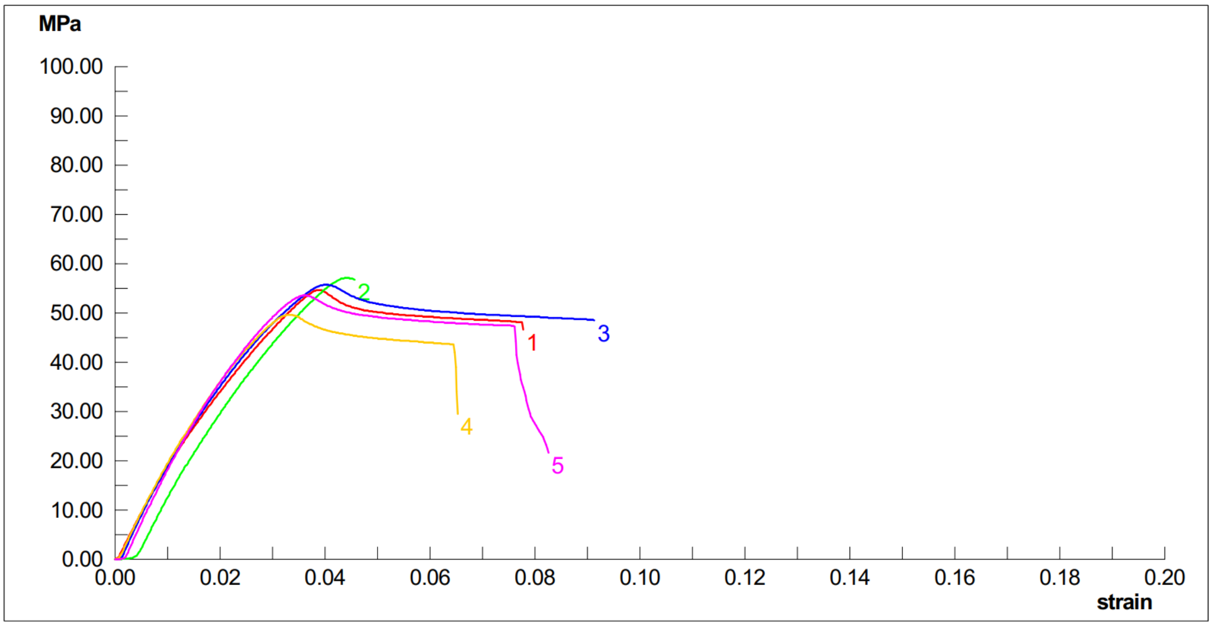

Figure S5: Stress–strain curves of tensile testing of GO-SG-ZH/PLA films.

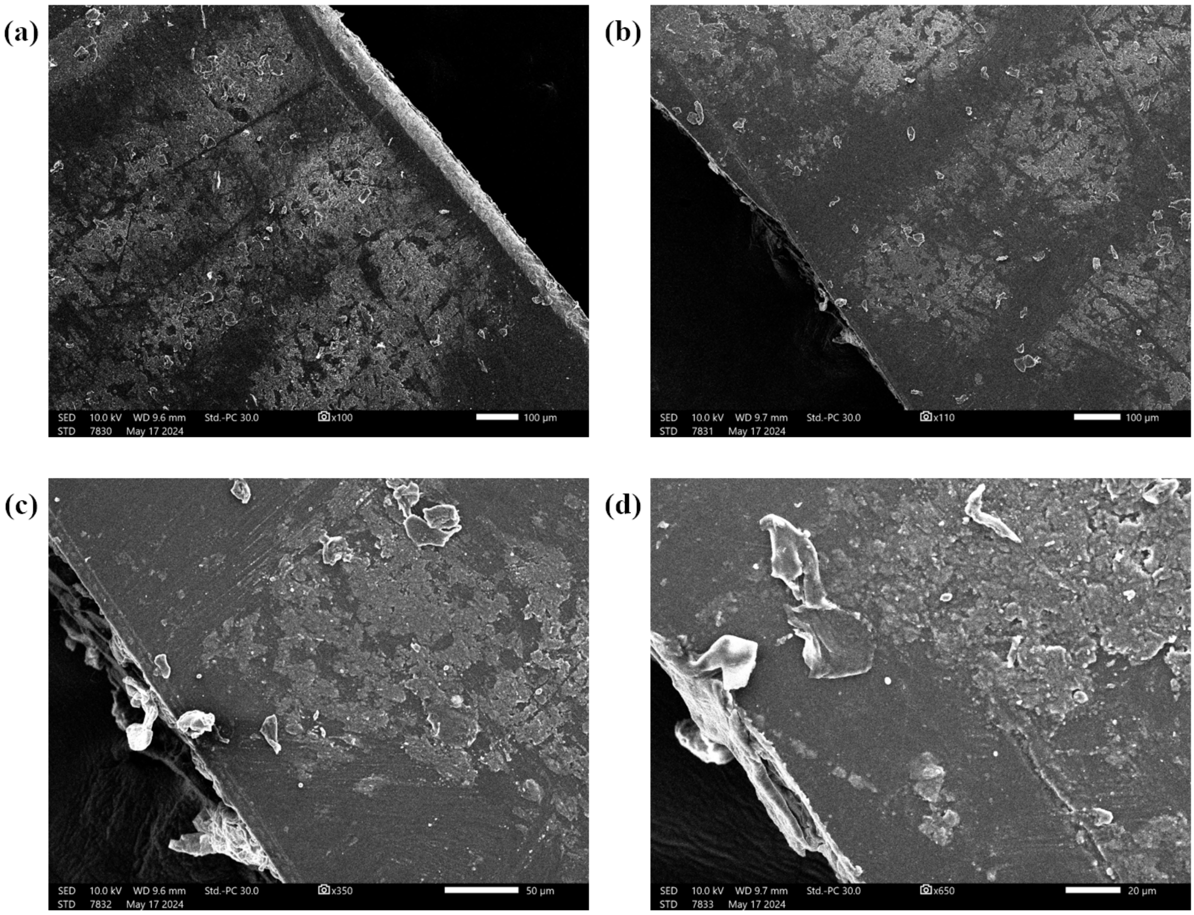

Figure S6: SEM images of surfaces and fractures of GO-SG-ZH/PLA films after tensile testing. The fractures were generated by the tensile measurement.
